# Essential oil-grafted copper nanoparticles as a potential next-generation fungicide for holistic disease management in maize

**DOI:** 10.3389/fmicb.2023.1204512

**Published:** 2023-07-06

**Authors:** Lham Dorjee, Robin Gogoi, Deeba Kamil, Rajesh Kumar, Tapan Kumar Mondal, Sudeepta Pattanayak, Bishal Gurung

**Affiliations:** ^1^Division of Plant Pathology, ICAR-Indian Agricultural Research Institute, New Delhi, India; ^2^Division of Agricultural Chemicals, ICAR-Indian Agricultural Research Institute, New Delhi, India; ^3^Division of Molecular Biology and Biotechnology, ICAR-Indian Agricultural Research Institute, New Delhi, India; ^4^Division of Forecasting and Agricultural Systems Modelling, ICAR-Indian Agricultural Statistics Research Institute, New Delhi, India

**Keywords:** essential oil-grafted CuNPs, maize, phytopathogens, physiological traits, MLB disease, management

## Abstract

Sustainable food production is necessary to meet the demand of the incessantly growing human population. Phytopathogens pose a major constraint in food production, and the use of conventional fungicides to manage them is under the purview of criticism due to their numerous setbacks. In the present study, essential oil-grafted copper nanoparticles (EGC) were generated, characterized, and evaluated against the maize fungal pathogens, *viz., Bipolaris maydis, Rhizoctonia solani* f. sp. *sasakii, Macrophomina phaseolina, Fusarium verticillioides*, and *Sclerotium rolfsii*. The ED_50_ for the fungi under study ranged from 43 to 56 μg ml^−1^, and a significant inhibition was observed at a low dose of 20 μg ml^−1^ under *in vitro* conditions. Under net house conditions, seed treatment + foliar spray at 250 and 500 mg L^−1^ of EGC performed remarkably against maydis leaf blight (MLB), with reduced percent disease index (PDI) by 27.116 and 25.292%, respectively, in two *Kharif* seasons (May-Sep, 2021, 2022). The activity of enzymatic antioxidants, *viz.*, β-1, 3-glucanase, PAL, POX, and PPO, and a non-enzymatic antioxidant (total phenolics) was increased in treated maize plants, indicating host defense was triggered. The optimum concentrations of EGC (250 mg L^−1^ and 500 mg L^−1^) exhibited improved physiological characteristics such as photosynthetic activity, shoot biomass, plant height, germination percentage, vigor index, and root system traits. However, higher concentrations of 1,000 mg L^−1^ rendered phytotoxicity, reducing growth, biomass, and copper bioaccumulation to high toxic levels, mainly in the foliar-sprayed maize leaves. In addition, EGC and copper nanoparticles (CuNPs) at 1,000 mg L^−1^ reduced the absorption and concentration of manganese and zinc indicating a negative correlation between Cu and Mn/Zn. Our study proposes that the CuNPs combined with EO (Clove oil) exhibit astounding synergistic efficacy against maize fungal pathogens and optimized concentrations can be used as an alternative to commercial fungicides without any serious impact on environmental health.

## 1. Introduction

To meet the demand of the incessantly growing population, which will reach approximately 9.7 billion by 2050 (United Nations, 2023), sustainable agriculture must be prioritized for food and nutritional security. Maize (*Zea mays* L.) is a wonder cereal crop, serving as a staple food in many countries, and it is endowed with high nutritional value. In addition, maize also contains phytochemicals such as carotenoids and anthocyanins (Siyuan et al., 2018), which have an undeniable role as anti-carcinogenic, antidiabetic, antimicrobial, and anti-inflammatory. Phytosterols, rich in maize, also have myriads of health benefits, such as lowering the absorption of cholesterol (Bae et al., 2022). Furthermore, it also finds its utility in biofuel production, animal feed, forage, and chemical production. Although the versatility and adaptability of maize as a crop are unmatched, fungal diseases are major constraints in its production. Important maize diseases such as maydis leaf blight (MLB), banded leaf sheath blight (BLSB), charcoal rot, Curvularia leaf spot (CLS), Turcicum leaf blight (TLB), Fusarium stalk rot (FSR), downy mildew (DM), and southern corn rust, render yield losses of approximately 80–100% (Bruns, 2017), 11–40%, and even up to 100% under favorable condition (Izhar and Chakraborty, 2013; Gao et al., 2014), 25–32% (Krishna et al., 2013), 10–60% (Akinbode, 2010), 13–50% (Tefferi et al., 1996), 10–42% (Harlapur et al., 2002), 20–30% (Spencer and Dick, 2002), and 80–84% (Reyes, 1953), respectively. In addition to fungal diseases, bacterial stalk rot, maize dwarf mosaic, and maize rough dwarf also cause loss of up to 85% (Thind and Payak, 1985), 90% (Goldberg and Brakke, 1987), and 10–70% (Dovas et al., 2003), respectively.

The application of chemical fungicides in the field is inevitable to relieve crops from pathogenic stress and other pest attacks. However, fungicides have earned their infamy for having severe consequences. They are proven to have adverse effects on non-target organisms and the environment due to their high toxicity, non-biodegradable nature, and long residual activity (Kamal et al., 2020). Moreover, systemic fungicides lead to the development of fungicidal resistance due to their specific site of action. In addition, fungicides take a toll on the farmers due to their high cost. Hence, conventional methods need to be reoriented toward more sustainable disease management.

Nanoscience, a promising and blossoming science, finds applications in various fields, including agriculture (Sharma et al., 2021). Any material would be considered a nanoparticle and would come under the study of nanotechnology if it is within the size range of 0.1 to 100 nanometers (Harish et al., 2022). In the field of agriculture, nanoscience has contributed to the development of varieties of crops by using nanoparticles as mutagens, and the improvement of fertilizer's efficiency by using a slow release of nanomaterial-assisted fertilizers, micronutrients, and bio-fertilizers (Usman et al., 2020; El-Saadony et al., 2021). In crop protection, the delivery of pesticides by the controlled release by encapsulating in nanomaterials and directly using nanoparticles as fungicides or bactericides has found its profound utility (Jampílek and Králová, 2017). Nanoparticles (NPs) such as copper-based NPs have remarkable antimicrobial properties with high efficacy and durability due to the high surface area/volume ratio and many active sites on the surface (Crisan et al., 2021; Ermini and Voliani, 2021). In addition, NPs hold the potential for pathogen detection of various types (Khan et al., 2019; Tomer et al., 2021; Shivashakarappa et al., 2022). Essential oils are another important means of managing phytopathogens that effectively protect plants against pathogens and are considered environmentally benign. Essential oils are fragrant, volatile liquids synthesized by plants through complex metabolic pathways to protect themselves from phytopathogens (Rai et al., 2017). The significant and remarkable biological activities of essential oil against phytopathogens can be attributed to their lipophilic nature and low molecular weight, which render disruption of the cell membrane leading to cell death or inhibiting sporulation (Nazzaro et al., 2017). It contains one or two functional groups (aldehydes, esters, phenols, terpenes, phenol ethers, and alcohols) as key components which act as antimicrobial entities against a wide range of pathogens (Eze, 2016).

The combination of NPs and essential oil has been demonstrated to exhibit synergistic antimicrobial potential which can effectively incapacitate pathogens (Rai et al., 2017). For instance, silver nanoparticles (AgNPs) in combination with cinnamon, citrus, and lavang essential oil exhibited synergistic antifungal and antibacterial against *Escherichia coli, Salmonella Typhimurium, Aspergillus niger, Penicillium chrysogenum*, and *Mucor circinelloides* (Begum et al., 2022). Casein NPs combined with eugenol exhibited excellent bactericidal efficacy against *Staphylococcus aureus and Bacillus sp*. (Wang et al., 2021). AgNPs and *oregano* essential oil against different gram-positive and gram-negative bacteria showed excellent synergistic efficacy (Scandorieiro et al., 2016). In addition, thyme and cinnamon essential oil encapsulated in chitosan nanoparticles exhibited remarkable antimicrobial properties against *Enterococcus sp., E. coli, Klebsiella pneumonia*, and *Pseudomonas aeruginosa* (Barrera-Ruiz et al., 2020). Nevertheless, the efficacy of the combination of CuNPs and clove oil against phytopathogens has not been reported hitherto.

In the present study, our objective was to develop an alternative method for managing phytopathogens, focusing on maize fungal pathogens. We, therefore, developed EO-grafted CuNPs with CuNPs and EO (clove) oil as the key components which exhibited significant antifungal activity *in vitro* and *in vivo*. Furthermore, the possible roles of the combination of CuNPs and EO (clove oil), i.e., EGC, CuNPs, and EO, in triggering defense enzymatic and non-enzymatic antioxidants in maize crops were studied. In addition, various phytotoxic impacts on maize plants due to EGC, CuNPs, and EO (clove oil) have been determined.

## 2. Materials and methods

### 2.1. Synthesis of CuNPs and preparation of EO-grafted CuNPs

Copper nanoparticles were synthesized by a modified protocol (Dorjee et al., 2023). Analytical grades of copper sulfate (CuSO_4_), sodium borohydride (NaBH_4_), ascorbic acid (C_6_H_8_O_6_), and polyethylene glycol 8000 (PEG 8000) were employed. CuSO_4_ (0.1 M) solution of 100 ml and 20 ml of 0.02M PEG 8000 was mixed and stirred for over 1 h using a magnetic stirrer (Hot stirrer, REXIM RSH-1D, China). An ascorbic acid (0.5 M) of 40 ml was added, allowed to stir at 500 rpm for 30 min with heating at 100°C followed by gradual addition of 80 ml of NaBH_4_. The pH of the reaction was adjusted to 6.5 to 7 by adding 1N NaOH. The essential oil under study, clove oil (EO), having eugenol as the major component of the premium grade was used (Clove oil extra pure, SRL, Mumbai, India), which was supposedly grafted on synthesized CuNPs by adding 40 ml of EO (10,000 ppm) to the 200 ml of CuNPs with a constant stirring at 500 rpm for 30 min using a magnetic stirrer (Hot stirrer, REXIM RSH-1D, China). The concentration was optimized such that the stock formulation had 2,000 ppm of CuNPs and EO.

### 2.2. Characterization

#### 2.2.1. UV-Vis spectroscopy

The optical absorption spectra of EO-grafted CuNPs were analyzed using an ultraviolet–visible spectrophotometer (Hitachi, U-3900, Version 2J2530004, Japan) with a regular interval of 0.5 nm in the range of 300 to 800 nm wavelength.

#### 2.2.2. X-ray diffraction analysis

For the X-ray diffraction analysis, EO-grafted CuNPs were harvested by centrifuging at 12,000 rpm for 5 min and then allowing them to dry at room temperature. The powdered sample was subjected to XRD (Bruker, D2 phase, Germany) with kα radiation (λ = 1.5406 Å), accelerating voltage/scanning speed of 3° per min, an operating voltage of 30 kV, and scanning speed in the 2θ range of 30 to 80°. Furthermore, the average particle size was evaluated by using the formula given by Scherrer.


$$\begin{array}{l} D=\frac{K\lambda }{\beta cos\theta } \end{array}$$

where D is the size of the particle, K is the Scherrer's constant also known as dimensionless shape factor (K = 0.94), λ is the wavelength of the x-ray (1.5406 Å), β is full width at half maximum (FWHM) of the diffraction peak (calculated by using Origin pro, 2023), and θ is the angle of the diffraction or Bragg angle (Patterson, 1939). The d-spacing value was calculated by using Bragg's equation.


$$\begin{array}{l} d=\frac{n\lambda }{2sin\theta }\; \end{array}$$

where d is the spacing between diffracting planes, n is an integer, λ is the beam wavelength, and θ is the incident angle. Subsequently, the crystal structure was determined by using Panalytical X'Pert High Score software.

#### 2.2.3. Dynamic light scattering and zeta potential analysis

The hydrodynamic size, particle size distribution, polydispersity index, and zeta potential to ascertain the stability of EGC were determined using the Zetasizer Nano series (Malvern Panalytical, UK). A suspension of EGC was ensured for its homogeneity, and the instrument was adjusted for laser and detector settings as per the manufacturer's instructions. The sample was sonicated for 20 min and transferred to a cuvette or sample cell, ensuring the sample was free of air bubbles and was placed in the instruments keeping the temperature at 25°C and backscatter angle at 173 degrees. The data were collected and further interpreted.

#### 2.2.4. Transmission electron microscopy

To determine the morphology of CuNPs, TEM (Jeol 1011 100 kV, Japan) was employed. A 40-min sonicated sample was placed on a 400-mesh carbon-coated copper grid and then stained with 2% uranyl acetate. Before the observation, the sample was left at room temperature for drying for 1 h. Furthermore, the average particle size was calculated by using “ImageJ and Origin Pro, 2023” software.

#### 2.2.5. Fourier transform infrared spectroscopy

The synthesized EO-grafted CuNPs were subjected to FTIR spectroscopy (Bruker, Tensor 37, Germany) to determine the functional groups present in the formulation. The sample preparation involved the addition of 100 mg of spectral-grade KBr to the sample followed by pressing under the high pressure of 6,000 kg cm^−2^ for about 2 min resulting in a translucent KBr pellet which was subjected to FTIR analysis. In total, 32 scans were made and the spectra of the sample were collected at a resolution and wave-number accuracy of 4 and 0.01 cm^−1^, respectively.

#### 2.2.6. Gas chromatography–mass spectrometry of essential oil-grafted CuNPs

The impact of CuNPs on EO (clove oil) was studied using GC-MS (Agilent Technologies, 8010C GC, USA) outfitted with an HP-5MS column (30 m × 0.25 mm; 0.25 mm) (Agilent Co., USA) coupled to a triple axis HED-EM 5975C mass spectrometer. Flow mode in split control was used to inject a volume of 2 μL at a ratio of 1:10. At a head pressure of 7.5 psi, the carrier gas flow was set at 0.75 mL min^−1^ helium (High purity >99.99%, New Delhi, India). The oven's temperature was initially set at 40°C for 1 min and then was raised with a gradient of 3°C min^−1^ to reach 60°C and held for 2 min. Furthermore, a 5°C min^−1^ gradient was used to bring the temperature to 220°C. Finally, the temperature increased to 300°C with an increment of 10°C min^−1^. The MS acquisition parameters were as follows: transfer line temperature 250°C, ion source 200°C, electron ionization 70 eV, full scan mode (50–550 mass units), and E.M voltage 1,220 V. The scan time was 1 s, and the compounds were identified by matching their respective mass spectra with the reference from the NIST (National Institute of Standards and Technologies) Mass Spectra Library.

### 2.3. Determination of antifungal activity *in vitro*

The efficacy of EGC *in vitro* was evaluated against five important maize fungal pathogens. *Bipolaris maydis* and *Rhizoctonia solani* f. sp. *sasakii* were isolated from the previously grown infected maize plant. Morpho-cultural identification was done and submitted to Indian Type Culture Collection, ICAR-IARI, New Delhi, with accession No. 9243 and 9244, respectively. *Macrophomina phaseolina* and *Fusarium verticillioides* were obtained from Maize Pathology Laboratory. *Sclerotium rolfsii* (Accession No. 8383) was received from Indian Type Culture Collection, ICAR-IARI, New Delhi. The poisoned food technique was used (Nene and Thapliyal, 1979) to evaluate the different concentrations of EGC. Except for *B. maydis*, potato dextrose agar (PDA) was used as a growth media containing 20% meshed potato, 2% dextrose, and 2% agar (bacterial grade, SRL). For *B. maydis*, healthy maize leaf extract was used, added with 2% sucrose and 2% agar (Bacterial grade, SRL). The media were sterilized by using an autoclave at 15 psi, 121°C for 20 min. The activity was carried out in a UV-sterilized laminar airflow cabinet. To the media, EGC of different concentrations from 20 to 140 μg ml^−1^ was added using a pipette. Media was poured into sterilized Petri plates, allowed to solidify, and inoculated with a mycelial disk of 5 mm using a flame-sterilized inoculation loop, and then, the plates were sealed with parafilm strip. Incubation of the fungi under study was done at 28 ± 2°C with a relative humidity of 60 ± 5%. Commercial fungicides (carbendazim 50% WP, hexaconazole 5% EC, and mancozeb 45% WP), CuNPs, and EO (clove oil) at 100 and 200 μg ml^−1^ were used for comparison. The radial growth was measured after the control plate attained full growth, and the inhibition percentage was calculated using the Vincent (1947) formula.


$$I=(C-\frac{T}{T})\times 100$$

where I is the percent inhibition, C is radial growth/diameter in control (cm), and T is radial growth/diameter in treatment (cm).

### 2.4. Determination of ED_50_ value

The ED_50_, i.e., the median effective dose of the EGC, was determined by using a series of different concentrations (20–140 ug ml^−1^). Percent inhibition was calculated which was further used to calculate “corrected percent inhibition (CPI)” by the formula described by Abbott.


$$CPI=\frac{IP-CF}{100-CF}\times 100\;$$

where IP is the percent inhibition (%), and CF is the correction factor calculated using the equation,


$$CF=\frac{D-CM}{CM}\times 100\;$$

where D is the diameter (cm) of the Petri plates, and CM is the mean diameter (cm) of fungal growth in control. Using the CPI data, probit analysis was carried out for their corresponding test concentrations and ED_50_ values in terms of μg ml^−1^ were computed using a statistical package for social sciences (SPSS version 16).

### 2.5. Determination of antifungal activity *in vivo* (net house)

The efficacy of the EGC was evaluated during the *Kharif* season (May–Sep) of 2021 and 2022 against the maydis leaf blight (MLB) disease caused by *Bipolaris maydis*. A susceptible variety of maize CM-119 was used for the study. A plot size of 14 × 7 m was used following appropriate agronomic practices. The spacing of 0.6 × 0.15 m (row × plant) was maintained with each row sown with eight maize plants. In total, 18 treatments including different combinations of EGC at 250, 500, and 1,000 mg L^−1^ *viz.*, seed treatment (ST), foliar spray (FS), seed treatment + foliar spray (ST+FS), negative control (only pathogen inoculated), absolute control (water spray), and commercial fungicides (Mancozeb 75% WP @ 2,000 mg L^−1^) with three replications in randomized block design (RBD) were studied. Sorghum seeds were used to prepare inoculum by overnight soaking, further sterilized for 3 consecutive days at 121°C, 15 lbs for 30 min. Three to four mycelia disks from the actively growing culture plate were dropped in the flasks followed by incubation at 28°C for 8 days. For uniform growth, periodic shaking was given. Completely grown fungus on seeds was subjected to air-drying for 4–5 days at room temperature and then ground to powder using a grinder machine. For the final use, the inoculum was mixed with sorghum seed ground powder in a 1:1 ratio. Whorl Inoculation (Payak and Sharma, 1983) was done on 35-day-old maize plants (stage 5 of maize growth stages) using 5 g of inoculum. Five days after inoculation (DAI), EGC at predetermined concentrations and Mancozeb 75% WP at recommended dose were sprayed on the plants. Disease scoring was performed two times on the 20th and 30th DAI using a modified 1–9 scale (Supplementary Table 1) adopted in the All India Coordinated Maize Improvement Project (AICMIP), 2016. The percent disease index (PDI)/severity was calculated by using the formula given by Mckinney (1923).


$$PDI=\frac{Sum\;of\;individual\;ratings}{Total\;No.ofplant\;assessed\times Maximum\;disease\;rating}\times 100\;$$

### 2.6. Biochemical study of enzymatic antioxidant

Maize leave samples were collected at five-time points after spraying (at 0, 24, 48, 72, and 96 h). The leaves were cut with sterilized scissors and froze immediately in liquid nitrogen and preserved at −80°C for further use. All the biochemical studies were carried out at 4°C.

#### 2.6.1. Peroxidase assay

POX activity was determined using guaiacol as a substrate. Standard protocol was followed (Castillo et al., 1984; Sarker and Oba, 2018) with required modifications elaborated in the Supplementary material. Enzyme activity was calculated as per the extinction coefficient from its oxidation product, i.e., tetra-guaiacol (€ = 26.6 mM^−1^cm^−1^), and expressed in terms of μM min^−1^g^−1^ fresh weight (FW).


$$POX\;activity\;(units\;per\;L)=\frac{\left(△Abs\times Total\;Assay\;Vol.\right)}{\left(△t\times \text{€}\times enzyme\;sample\;volume\right)}\;$$

where ΔAbs is the change in absorbance, Δt is the time of incubation (min), and € is the extinction coefficient.

#### 2.6.2. Polyphenol oxidase assay

The standard protocol described in the Supplementary material was followed to determine PPO activity (Mayer et al., 1966; Ashajyothi et al., 2020). PPO activity was ascertained by measuring the rate of quinone formation at 420 nm which increases over time. PPO activity was worked out as per the extinction coefficient from its oxidation product, pyrogallol (€ = 2.8 M^−1^ cm^−1^), and expressed in μM min^−1^ g^−1^ FW and was defined as the amount of enzyme that oxidized 1 μ mol of substrate min^−1^.


$$PPO\;activity\;(units\;per\;L)=\frac{\left(△Abs\times Total\;Assay\;Vol.\right)}{\left(△t\times \text{€}\times enzyme\;sample\;volume\right)}$$

where ΔAbs is the change in absorbance, Δt is the time of incubation (min), and € is the extinction coefficient.

#### 2.6.3. β-1, 3-glucanase assay

The effect of EGC on β-1, 3-glucanase activity was ascertained by following Abeles and Forrence (1970) method with slight modifications detailed in the Supplementary material. The activity was determined by using an equation derived from the D-glucose standard curve (Supplementary Figure 1) and expressed as μ mol g^−1^ FW. The equation for the assay is as follows:


y=0.0004x

where y is the absorbance, and x is the concentration calculated.

#### 2.6.4. Phenylalanine ammonia-lyase assay

To determine the effect of EGC on PAL activity, a standard method proposed by Aebi (1984) with requisite modifications was adopted using L-phenylalanine as a substrate. A detail of the protocol is given in the Supplementary material. The activity of PAL was calculated by using a standard curve of trans-cinnamic (Supplementary Figure 2) and expressed in terms of μg g^−1^ FW. The derived equation used for the calculation of PAL activity is given below.


y=0.0008x+0.0069 

where y is the absorbance, and x is the concentration calculated.

### 2.7. Determination of total phenolic content (non-enzymatic antioxidant)

The total phenolic content and its change upon treatment were determined following the protocol given by Ainsworth and Gillespie (2007) with requisite modification as described in the Supplementary material. Folin–Ciocalteu (FC) reagent was used for total phenol estimation using gallic acid as a standard (Ainsworth and Gillespie, 2007; Sarker and Oba, 2018). The standard curve graph (Supplementary Figure 3) was prepared using standard gallic acid prepared by mixing it in methanol, and the total phenol concentration was calculated using the regression equation derived from the standard graph. The final result was expressed as μg gallic acid equivalent (GAE) g^−1^ FW. The total phenol concentration (TPC) was calculated by using the following formula:


$$TPC=\frac{Y}{100}\;$$

where Y is the calibrated TPC (μg ml^−1^) equivalent to the gallic acid standard. Y was calculated using the following formula:


$$Y=\left(\frac{x}{EE}\right)\times TVE$$

where TVE is the total volume of enzyme extract, EE is the enzyme extract used for the assay, and x was calculated using the equation derived from the standard curve graph.


y=0.0063x+0.1147

where y and x indicate absorbance and concentration, respectively.

### 2.8. Phytotoxic studies

#### 2.8.1. Determination of physiological parameters of maize under EGC influence

A pot study was carried out to determine the effect of EGC on the maize plants (CM-119). Seeds were treated with different concentrations (six treatments) and each pot (Top radius = 12 cm, Base radius = 9, Height = 37 cm) was sown with seven seeds. A total of three replicated pots were maintained. Shoot length, root length, shoot biomass, root biomass, and the root–shoot ratio were measured as 35 DAS (days after sowing). Germination percentage and vigor index were calculated by using the following formula (Ali et al., 2021):


$$\begin{array}{l} Germination\;\left(\%\right)\;=\;\frac{No.\;of\;seed\;germinated}{No.\;of\;seeds\;sown}\times 100\;\\ \;Vigor\;Index\;=\;\left(Mean\;root\;length+Mean\;shoot\;length\right)\\ \;\times \;Germination\;(\%)\; \end{array}$$

For the root study, the roots were gently removed from the pots and dipped in running water and washed 2–3 times to achieve clean roots. The roots were stored at 4°C for further root system traits studies. The roots were scanned and analyzed to determine root morphological traits, *viz*., root length, root volume, and average diameter using WinRHIZO professional software (LA2400, Regent instrument, Quebec, Canada). The roots were then oven-dried at 65 ± 5°C for 72 h and weighed using an electronic balance (Sartorius, Germany) to determine the root biomass. Furthermore, the root length density was calculated by dividing the root length by soil volume (12,903 cm^3^) (Zhiipao et al., 2023).

#### 2.8.2. Histological observation

To ascertain the effect of EGC on guard cells of stomata, a histological assessment was performed using light microscopy (Olympus microscope connected to CMOS HDMI Truechrome HD, Radical, Japan). Leaf samples were collected 5 days after spraying from those lines which were seed-treated before sowing with different concentrations *viz.*, 250, 500, and 1,000 mg L^−1^. The leaf was cut into small pieces of 5 × 5 mm and dipped into a solution containing glacial acetic acid and absolute alcohol in a ratio of 1:1 overnight followed by incubation at 25 ± 2°C for 2 days. Furthermore, the leaf samples were observed under the microscope for anomalies or distortions rendered by EGC.

#### 2.8.3. Determination of effects of EO-grafted CuNPs on photosynthetic pigments

The chlorophyll contents (chlorophyll a, b, and total chlorophyll) of maize leaves exposed to EGC in the net house condition were estimated by using the dimethyl sulfoxide (DMSO) method (Hiscox and Israelstam, 1979; Barnes et al., 1992). Fully exposed and matured leaves (5th leaves from the ground) were collected and rinsed with sterile water. A sample of 0.5 g was weighed and immediately immersed in 10 ml of DMSO followed by incubation in a hot air oven (80°C) for 4 h. After the complete digestion, 1 ml of the extract was diluted with 5 ml DMSO, and the absorbance was documented using a spectrophotometer (Hitachi, U-3900, Version 2J2530004, Japan) at 645 and 663 nm wavelengths keeping DMSO as a blank. The chlorophyll content was calculated by using Arnon (1949) formula.


$$\begin{array}{r} Chlorophyll\;a\;\left(mg\;per\;g\;FW\right)\\ =\frac{\left\{\left(12.7\times OD663\right)-(2.69\times OD645)\times V\times DF\right\}}{1000\times W}\;\\ Chlorophyll\;b\;\left(mg\;per\;g\;FW\right)\\ =\frac{\left\{\left(22.7\times OD645\right)-(4.68\times OD663)\times V\times DF\right\}}{1000\times W}\;\\ Total\;chlorophyll\;\left(mg\;per\;g\;FW\right)\\ =\frac{\left\{\left(20.20\times OD645\right)-(8.02\times OD663)\times V\times DF\right\}}{1000\times W} \end{array}$$

where OD is the absorbance, V is the volume of DMSO used for the extraction of pigments, DF is the dilution factor, and W is the weight of the sample (g). The chlorophyll content was expressed in terms of mg g^−1^ FW.

The same extract was used for the estimation of carotenoid content under EGC stress (Kirk and Allen, 1965). The spectrophotometer reading (Hitachi, U-3900, Version 2J2530004, Japan) was recorded at 480 nm, and the carotenoid content was calculated using the following formula and expressed in terms of μg g^−1^ FW.


$$\begin{array}{r} Carotenoids\;\left(\text{μ}g\;per\;g\;FW\right)=OD480+\left(0.114\times OD663\right)\\ -(0.638\times OD645) \end{array}$$

where OD is the absorbance recorded at 480, 663, and 645 nm.

#### 2.8.4. Estimation of bioaccumulation of copper in maize leaves

The di-acid digestion method was used to determine the bioaccumulation of Cu in the maize leaves exposed to different treatments (net house condition) to ascertain whether the accumulation of Cu reaches to a phytotoxic level. The sample was collected 5 days after spraying, properly cleaned with deionized water, and dried to a constant weight. A 0.2 g of sample was immersed in concentrated HNO_3_ (10 ml) and left for 7–8 h for pre-digestion. Furthermore, in an acid-proof chamber, 10 and 2 ml of HNO_3_ and HClO_4_, respectively, were added followed by heating the Erlenmeyer flask on a hot plate for 1 h at 100°C and then raised the temperature to 200°C until the content turned colorless with white dense fumes. After the digestion, 30 ml of double-distilled H_2_O was added and filtered through Whatman No. 42 filter paper, and the volume was adjusted to 100 ml. The filtrate was subjected to atomic absorption spectroscopy (AAS) (Mortras Scientific, ADS1000 FSX, India), and the concentration of Cu in the maize leaves was calculated using the formula given below (Monni et al., 2000).


$$Cu\;concentration\;\left(mg\;per\;kg\;DW\right)=\frac{ASS\;reading\times DF}{W}$$

where DF is the dilution factor and W is the plant dry weight in g. The concentration of copper was expressed in terms of mg kg^−1^ dry weight (DW).

### 2.9. Statistical analysis

Experiments conducted in the present study were repeated twice. Complete randomized design (CRD) was used for *in vitro* (lab experiments) and *in vivo* (net house) experiment which was conducted by following randomized block design (RBD) with three replications. Statistical analysis of the data generated in the experiments was performed by following the procedure of SAS 9.4 (SAS Institute, 2003, Cary, NC). The significant difference between the treatments mean was determined by the Tukey HSD test (*p* ≤ 0.5) after analysis of variance (ANOVA) (Gomez and Gomez, 1984), and data in percentage were angular-transformed.

## 3. Result

### 3.1. Synthesis and characterization of EO-grafted CuNPs

**Figure 1:**
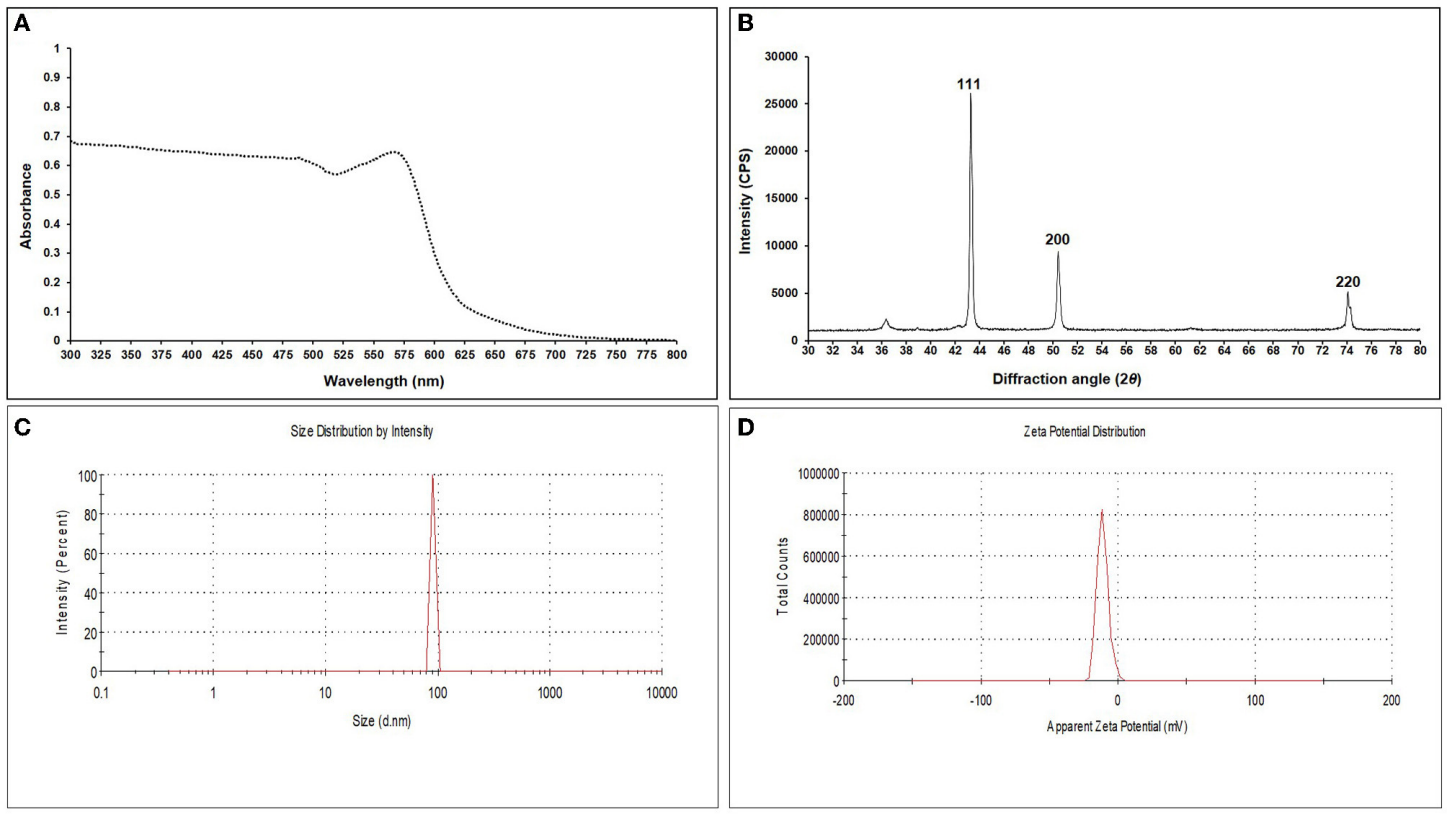
UV-Vis spectrum **(A)**, X-ray diffraction pattern **(B)**, DLS image exhibiting hydrodynamic size distribution **(C)**, and zeta potential **(D)** of synthesized essential oil-grafted CuNPs.

The addition of a reducing agent, ascorbic acid (C_6_H_8_O_6_), and sodium borohydride (NaBH_4_) led to the color change from blue to green and later to reddish yellow, respectively, indicating a reduction in particle size. For EGC, UV–visible spectra were recorded using the scanning mode in the range of 300–800 nm which revealed an absorption peak at 566 nm (Figure 1A). XRD analysis was carried out to ascertain the crystalline nature and size of synthesized EO-grafted CuNPs (Holder and Schaak, 2019). The X-ray data were recorded in the 2θ values range of 30° to 80°. The diffraction peaks were recorded at 43.299°, 50.437°, and 74.139° which correspond to miller indices (*hkl*) 111, 200, and 220, respectively, indicating face-cubic structure (FCC) of EGC (Figure 1B). The *d*-spacing value was found to be 2.039, 1.750, and 1.191 Å, respectively, which was calculated using the 2θ value achieved from the raw data. By employing the Scherrer formula, the average size of crystallite particles was found to be 29.208 nm (Supplementary Table 2). DLS studies revealed an average particle size of 91.28 nm with a polydispersity index of 1 (Figure 1C). The zeta potential, i.e., the surface charge of particles, was found to be −21.5 mV (Figure 1D).

**Figure 2:**
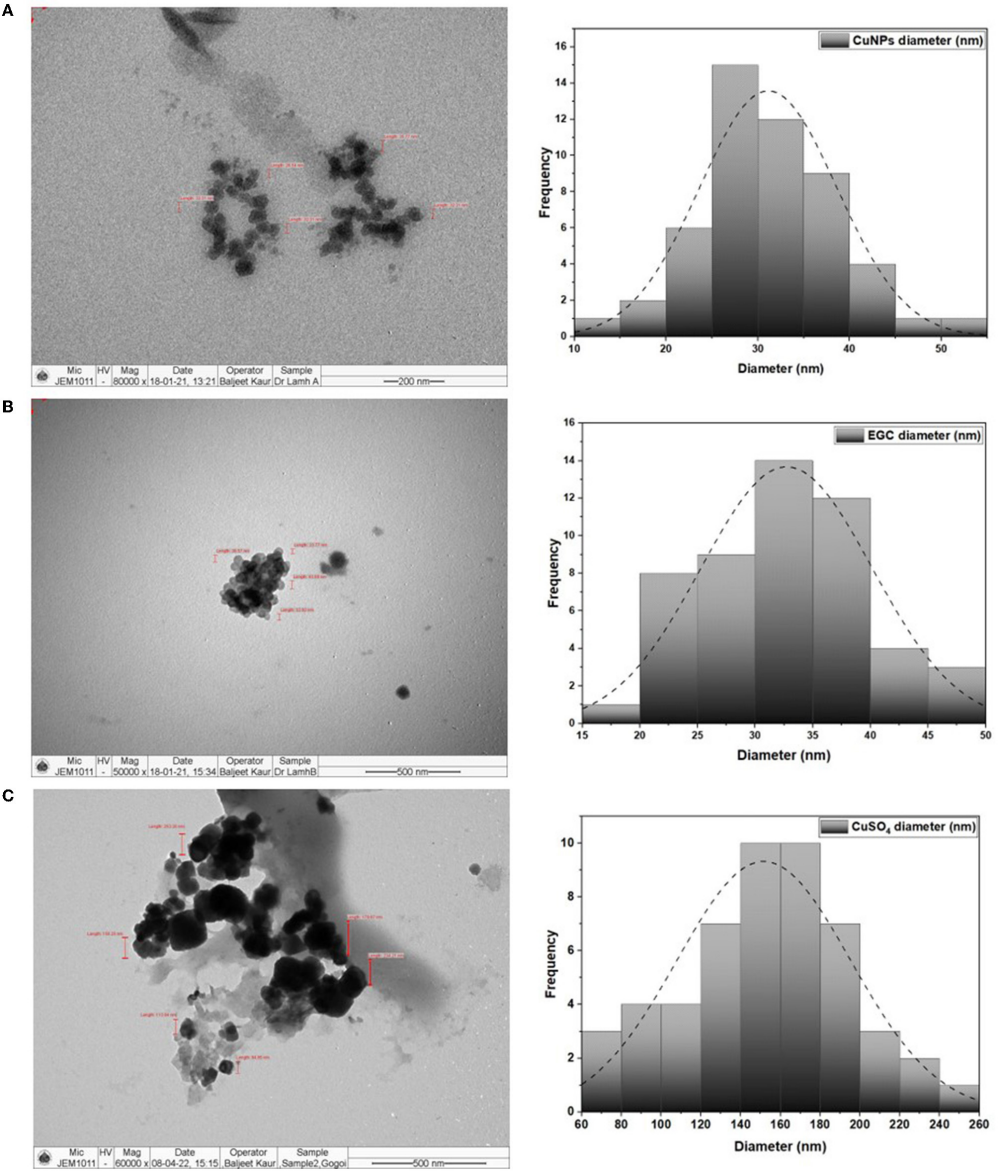
TEM image and size distribution of CuNPs **(A)**, essential oil-grafted CuNPs **(B)**, and CuSO_4_ **(C)** at 50000X magnification.

**Figure 3:**
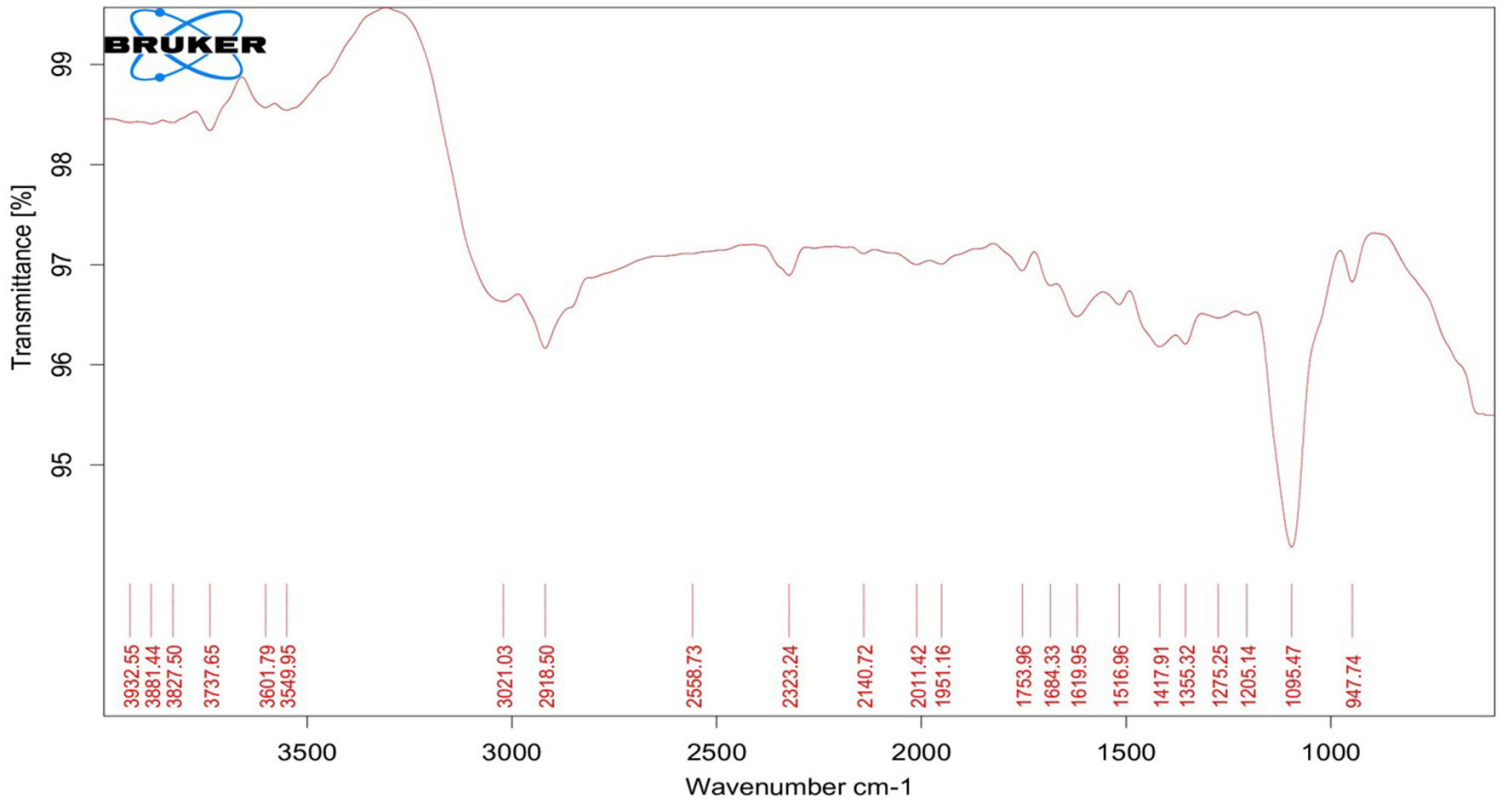
Fourier transform infrared spectroscopy spectrum of essential oil-grafted CuNPs.

**Figure 4:**
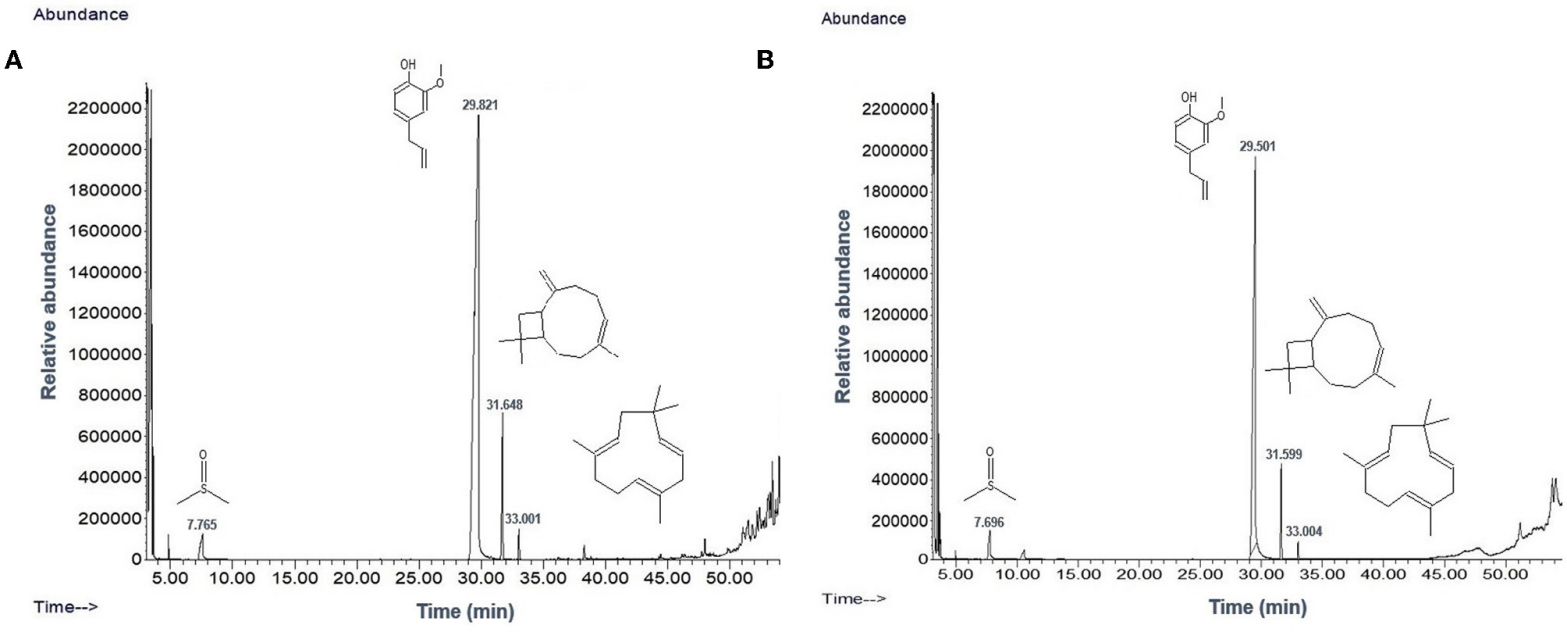
Chromatogram of EO (clove oil) **(A)** and EO+CuNPs **(B)** at 2,000 μg ml^−1^ indicating no adverse effect of CuNPs on EO.

TEM analysis was carried out for three different samples, *viz.*, CuNPs, EGC, and CuSO_4_. TEM analysis measured spherical CuNPs of size 25–90 nm (average size = 30.539 nm) (Figure 2A) on a carbon grid, whereas grafting with essential oil led to some extent of agglomeration with spherical nanoparticles of size ranging from 23 to 85 nm (average size = 38.478 nm) (Figure 2B). For comparison, TEM analysis of the CuSO_4_ solution was done, which revealed an average particle size of >180 nm (Figure 2C). FTIR spectra showed significant peaks at 3,737, 2,918, 1,417, 1,619, 1,684, 1,753, 1,095, 2,323, and 2,011 cm^−1^. The IR spectra also revealed a shift of peaks at 3,021, 1,516, and 1,355 cm^−1^ (Figure 3). Gas chromatography–mass spectrometry (GC-MS) revealed the presence of 69.66% of eugenol in a sample containing CuNPs + EO (Clove oil) (Figure 4B) which was comparable to 72.40% eugenol present in the sample with only EO (Clove oil) (Figure 4A) indicating the main constituents, i.e., eugenol remains intact in the presence of metal nanoparticles. Since DMSO was used to dissolve the EO, its presence was observed in the proportion of 4.23 and 3.95%, respectively. Apart from aforesaid constituents, trans-caryophyllene and α-caryophyllene were also detected (Table 1).

**Table 1:** GC-MS analysis of EO and CuNPs+ EO.

**Peak**	**EO**			**CuNPs**+**EO**		
	**Compounds**	**RT (min)** ^*^	**PA (%)** ^#^	**Compounds**	**RT (min)** ^*^	**PA (%)** ^#^
1	Eugenol	29.821	72.40	Eugenol	29.501	69.66
2	DMSO	7.765	3.95	DMSO	7.696	4.03
3	Trans-Caryophyllene	31.648	19.50	Trans-Caryophyllene	31.599	18.48
4	α-caryophyllene	33.001	3.67	α-caryophyllene	33.004	3.82

^*^Retention time. ^#^Peak area percentage in relation to peak total area.

### 3.2. *In vitro* efficacy of EO-grafted CuNPs on maize fungal pathogens

**Figure 5:**
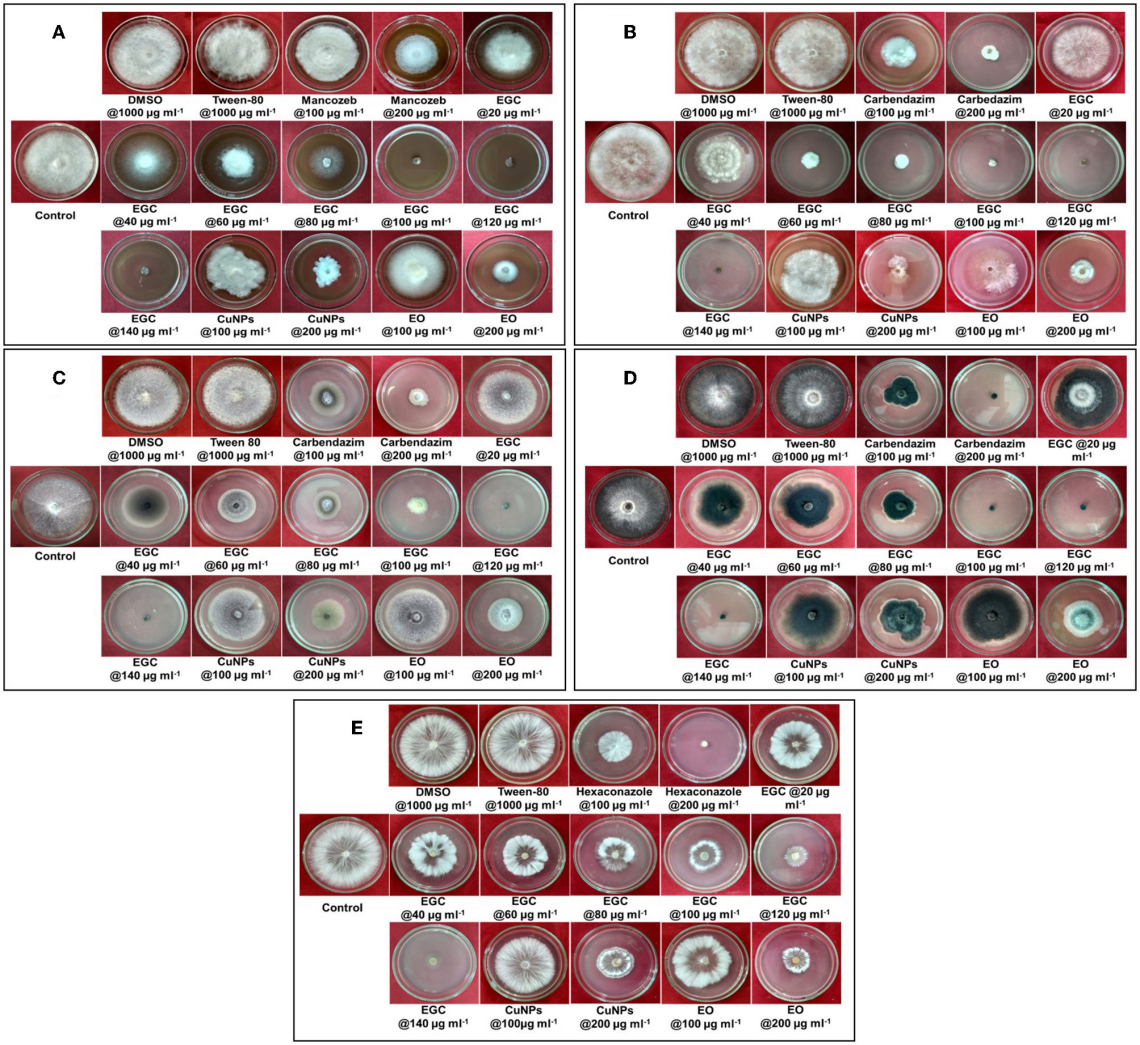
Concentration-dependent radial growth reduction in *Bipolaris maydis* **(A)**, *Rhizoctonia solani* f.sp. *sasakii* **(B)**, *Fusarium verticillioides* **(C)**, *Macrophomina phaseolina* **(D)**, and *Sclerotium rolfsii* **(E)** *in vitro* exposed to essential oil-grafted CuNPs.

**Figure 6:**
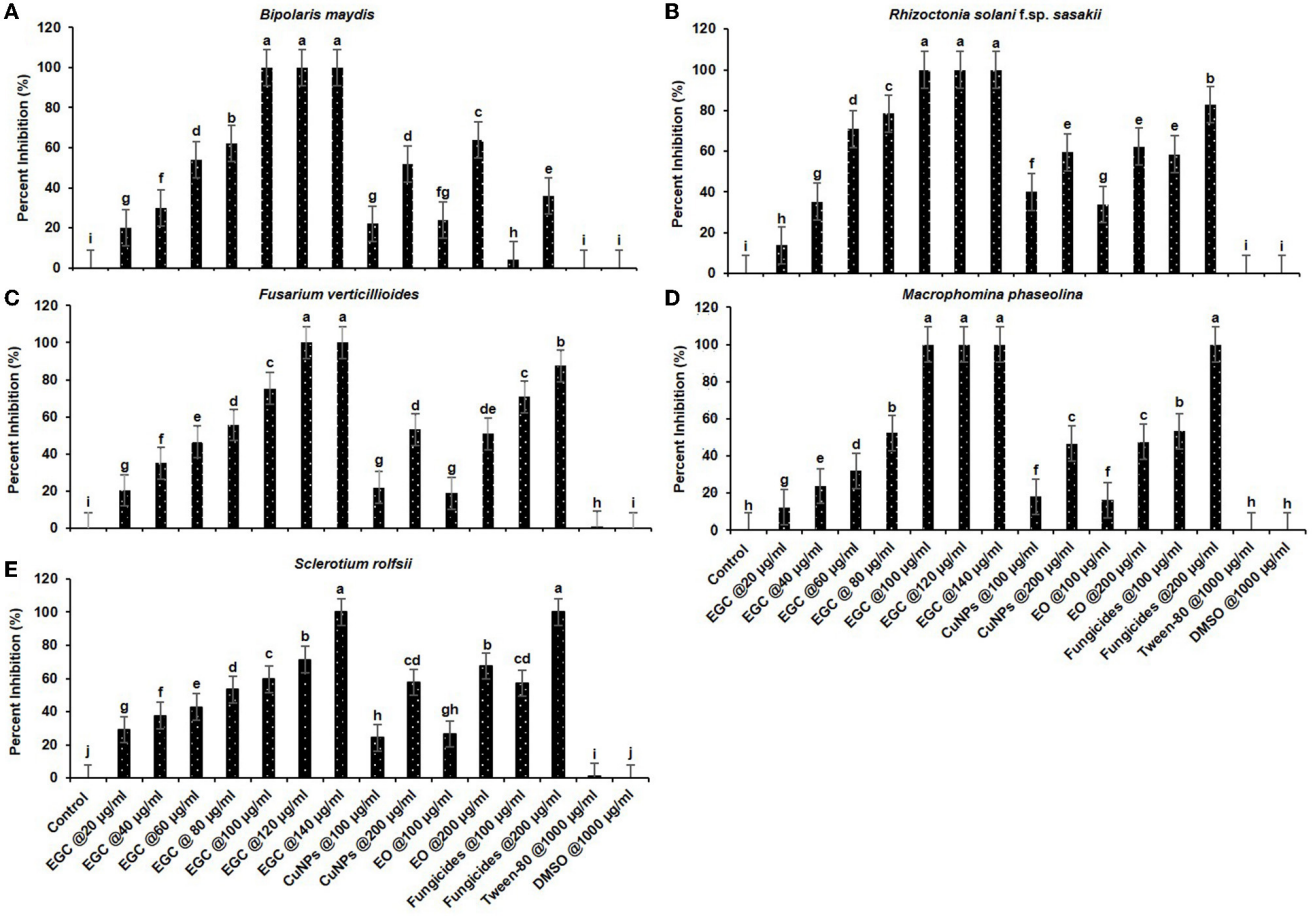
*In vitro* efficacy of essential oil-grafted CuNPs on *Bipolaris maydis* **(A)**, *Rhizoctonia solani* f.sp. *sasakii* **(B)**, *Fusarium verticillioides* **(C)**, *Macrophomina phaseolina* **(D)**, and *Sclerotium rolfsii* **(E)** *in vitro* exposed to EGC. Data (mean ± standard errors) with different letters are significant in each graph (Tukey, HSD, *p* ≤ 0.01). EGC, essential oil-grafted CuNPs; CuNPs, copper nanoparticles; EO, essential oil (clove oil); fungicides, mancozeb 75% WP against B. *maydis*, carbendazim 50% WP against R. *solani* f.sp. *sasakii*, F. *verticillioides*, and M. *phaseolina*, and hexaconazole 5% SC against S. *roflsii*.

Significant inhibition was achieved against the five different fungi at a concentration of 20 μg ml^−1^, and complete inhibition was observed at 100 μg ml^−1^ for the fungi under study (*p* ≤ 0.01) (Figures 5, 6; Supplementary Tables 3, 4). In the case of *B. maydis*, the result indicated a synergistic effect of EGC (containing CuNPs+EO), and it performed better than mancozeb 75% WP at 100 μg ml^−1^ (Figures 5A, 6A; Supplementary Table 3). Similarly, for the other three fungi, *viz., R. solani*. f. sp. *sasakii, M. phaseolina*, and *F. verticillioides*, a synergistic effect was also observed and the inhibition at 100 μg ml^−1^ EGC was significantly higher than that of carbendazim used at the same concentration (*p* ≤ 0.01). In the case of *R. solani*. f. sp. *sasakii*, EGC at 60 μg ml^−1^ performed better than the 100 μg ml^−1^ of carbendazim (Figures 5B, 6B; Supplementary Table 3). At higher concentrations (80 μg ml^−1^), a change in the morphology and color was observed in *F. verticillioides* (Figures 5C, 6C). Complete inhibition of radial growth was observed at 140 μg ml^−1^ for *S. roflsii*. However, an EGC concentration of 120 μg ml^−1^ was at par with 100 μg ml^−1^ of hexaconazole (Figures 5E, 6E; Supplementary Table 4). Since DMSO and Tween-80 were used to dissolve the EO, based on the final concentration added to media for bioassay of aforesaid compounds, 1,000 μg ml^−1^ of each was used to ascertain its effect. Both the compounds did not exhibit an inhibitory effect at the concentration studied against the fungi.

### 3.3. ED_50_ value of EO-grafted CuNPs

The probit analysis was performed to determine the effective dose causing 50% inhibition (ED_50_) by EGC against five different important maize pathogens. For *B. maydis* and *R. solani* f.sp. *sasakii*, ED_50_ was perceived as 46.900 and 43.208 μg ml^−1^, respectively. In the case of *F. verticillioides, S. roflsii*, and *M. phaseolina*, ED_50_ was found as 51.003, 55.096, and 56.499 μg ml^−1^, respectively (Table 2).

**Table 2:** ED_50_ estimated by probit analysis for essential oil-grafted CuNPs.

**Target fungi**	**ED_50_ (μg ml^−1^)**	**Fiducial limit (**μ**g ml**^**−1**^**)**		**Chi-square (χ^2^)**	**Regression equation**
*B. maydis*	46.900	23.709	67.924	71.944	*y* = 2.0 × *x*+(−3.600)
*R. solani* f.sp. *sasakii*	43.208	32.267	53.265	27.572	*y* = 3.6 × *x*+(−6.143)
*F. verticillioides*	51.003	26.728	73.843	56.633	*y* = 2.9 × *x*+(−4.714)
*S. rolfsii*	55.096	19.080	93.846	45.355	*y* = 1.2 × *x*+(−2.040)
*M. phaseolina*	56.499	24.197	89.507	110.389	*y* = 2.0 × *x*+(−3.800)

### 3.4. *In vivo* efficacy of EO-grafted CuNPs against maydis leaf blight

**Figure 7:**
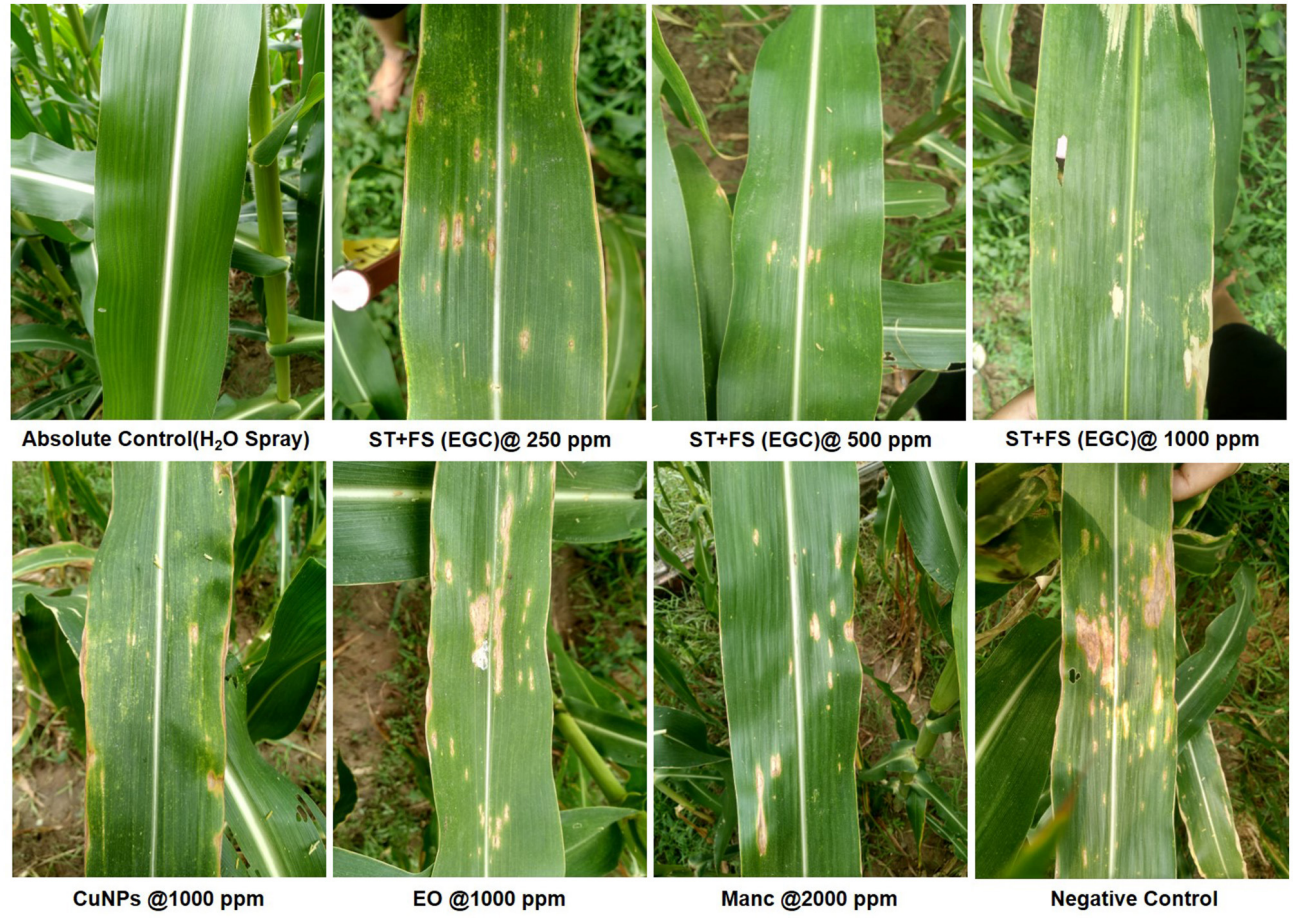
Maydis leaf blight (MLB) symptoms on artificially inoculated maize plants exposed to different treatments. EGC, essential oil-grafted CuNPs; CuNPs, copper nanoparticles; EO, essential oil (Clove oil); Manc, mancozeb 75% WP; ST, seed treatment; FS, foliar spray; negative control, only pathogen inoculated.

*In vivo* data were recorded two times, namely 20 and 30 DAI in two different *Kharif* seasons (May–September) of 2021 and 2022 (Supplementary Tables 5, 6). All the maize plants were inoculated with *B. maydis* inoculum 35 DAS except for absolute control. Out of ten seed treatments (ST), five were complemented with foliar spray (FS) five DAI. Among the different treatments under study, ST+FS (EGC) at 1,000 mg L^−1^ performed best with the least PDI of 20.635 and 23.544% in the 1^st^ and 2^nd^ scoring, respectively (Figure 7; Table 3). However, a concentration of 1,000 mg L^−1^ either as spray or seed treatment resulted in phytotoxicity. Moreover, the ST of EGC and EO (clove oil) at 1,000 mg L^−1^ compromised the germination and growth of the plant. Other combinations of EGC also rendered significantly less PDI as compared to the negative control (64.550% and 68.519% in the 1st and 2nd scoring, respectively) (*p* ≤ 0.05) (Figure 7; Table 3). Seed treatment at 250 and 500 mg L^−1^ showed satisfactory results with less PDI in both the 1^st^ and 2^nd^ scoring. Nevertheless, ST (EGC) or FS (EGC) at 250 and 500 mg L^−1^ were at par statistically (*p* ≤ 0.05). FS of EGC led to slightly higher PDI as compared to the seed-treated lines in both scorings. PDI in maize plants treated with ST+FS (EGC) at 250 and 500 mg L^−1^ was significantly less, and comparatively, ST+FS (EGC) at 500 mg L^−1^ performed better. As compared to mancozeb 75% WP sprayed at 2,000 mg L^−1^, the PDI of plants treated with ST+FS (EGC) at 250, 500, and 1,000 mg L^−1^, ST (EGC) at 250 and 500 mg L^−1^, and FS (EGC) at 1,000 mg L^−1^ was significantly lower proving the better performance over the commercial fungicides (*p* ≤ 0.05). CuNPs in combination with EO exhibited a synergistic effect, yet EO alone was feeble in reducing the PDI. Nonetheless, the PDI in maize plants treated with EO was comparatively less as compared to the negative (*p* ≤ 0.05). In all the treatments, the increase in the PDI between two scoring with 10 days gap was only 5–7%, but in the case of negative control, a drastic increase in the PDI was observed (Table 3).

**Table 3:** *In vivo* efficacy (Net house) of EO-grafted CuNPs against maydis leaf blight of maize.

**S. No**.	**Treatments**	**1st scoring**		**2nd scoring**	
		**Score** ^*^	**PDI (%)** ^*^	**Score** ^*^	**PDI (%)** ^*^
1	ST (EGC) @250 mg L^−1^	2.762 ± 0.024^c^	30.688 (33.626 ± 0.164)^*# de*^	2.976 ± 0.024^cde^	33.069 (35.089 ± 0.161)^de^
2	ST (EGC) @500 mg L^−1^	2.619 ± 0.086^cd^	29.101 (32.627 ± 0.599)^ef^	2.786 ± 0.071^cde^	30.952 (33.787 ± 0.490)^ef^
3	ST (EGC) @1,000 mg L^−1^	2.238 ± 0.063^cd^	24.868 (29.896 ± 0.463)^g^	2.476 ± 0.063^cde^	27.513 (31.620 ± 0.450)^g^
4	ST (CuNPs) @1,000 mg L^−1^	2.619 ± 0.086^cd^	29.100 (32.627 ± 0.599)^ef^	2.810 ± 0.063^cde^	31.217 (33.951 ± 0.432)^ef^
5	ST (EO) @100 mg L^−1^	4.500 ± 0.082^b^	50.000 (44.982 ± 0.525)^c^	4.786 ± 0.109^b^	53.174 (46.802 ± 0.695)^c^
6	FS (EGC) @250 mg L^−1^	3.048 ± 0.063^c^	33.862 (35.569 ± 0.424)^d^	3.262 ± 0.024^c^	36.243 (37.000 ± 0.158)^d^
7	FS (EGC) @500 mg L^−1^	3.000 ± 0.041^c^	33.333 (35.249 ± 0.278)^d^	3.119 ± 0.024^cd^	34.656 (36.050 ± 0.159)^de^
8	FS (EGC) @1,000 mg L^−1^	2.643 ± 0.124^cd^	29.365 (32.788 ± 0.865)^ef^	2.857 ± 0.109^cde^	31.746 (34.273 ± 0.743)^e^
9	FS (CuNPs) @1,000 mg L^−1^	2.857 ± 0.071^c^	31.746 (34.277 ± 0.490)^de^	3.071 ± 0.071^cd^	34.126 (35.728 ± 0.481)^de^
10	FS (EO) @1,000 mg L^−1^	5.071 ± 0.180^ab^	56.349 (48.633 ± 1.151)^b^	5.167 ± 0.156^b^	57.407 (49.245 ± 1.003)^b^
11	ST+FS (EGC) @250 mg L^−1^	2.357 ± 0.041^cd^	26.190 (30.767 ± 0.298)^fg^	2.524 ± 0.104^cde^	28.042 (31.953 ± 0.734)^fg^
12	ST+FS (EGC) @500 mg L^−1^	2.238 ± 0.086^cd^	24.868 (29.892 ± 0.635)^g^	2.429 ± 0.082^cde^	26.984 (31.277 ± 0.591)^g^
13	ST+FS (EGC) @ 1,000 mg L^−1^	1.857 ± 0.189^d^	20.635 (26.952 ± 1.476)^h^	2.119 ± 0.145^e^	23.544 (28.990 ± 1.091)^h^
14	ST+FS CuNPs) @1,000 mg L^−1^	2.238 ± 0.086^cd^	24.867 (29.892 ± 0.635)^g^	2.357 ± 0.109^de^	26.190 (30.758 ± 0.785)^gh^
15	ST+FS (EO) @1,000 mg L^−1^	4.571 ± 0.041^b^	50.794 (45.436 ± 0.262)^c^	4.714 ± 0.041^b^	52.381 (46.346 ± 0.263)^c^
16	Absolute control (Water Spray)	0.071 ± 0.041^e^	0.793 (4.115 + 2.147)^i^	0.167 ± 0.048^f^	1.851 (7.618 ± 1.255)^i^
17	Manc @2,000 mg L^−1^	3.199 ± 0.145^c^	34.656 (36.039 ± 0.970)^d^	3.310 ± 0.063^c^	36.772 (37.313 ± 0.415)^d^
18	NC (Pathogen only)	5.810 ± 0.063^a^	64.550 (53.439 ± 0.420)^a^	6.167 ± 0.126^a^	68.519 (55.857 ± 0.861)^a^

^*^Data are the mean of two different seasons with three replications. ^#^Data within the parentheses are angular-transformed values. Data (mean ± standard errors) followed by different letters in each column indicate a significant difference (Tukey HSD, *p* ≤ 0.05). Disease data scored twice on 20 and 30 DAI. ST, seed treatment; FS, foliar spray; PDI, percentage disease index; EGC, EO-grafted CuNPs; EO, essential oil (clove oil); Manc, mancozeb 75% WP; NC, negative control.

### 3.5. Biochemical responses to EO-grafted CuNPs

#### 3.5.1. Enzymatic antioxidants

Considering the high phytotoxicity of EGC and CuNPs at 1,000 mg L^−1^ and the observed detrimental effect of EO at 1,000 mg L^−1^ on germination and other growth parameters, the aforesaid treatments were not taken for biochemical studies.

**Figure 8:**
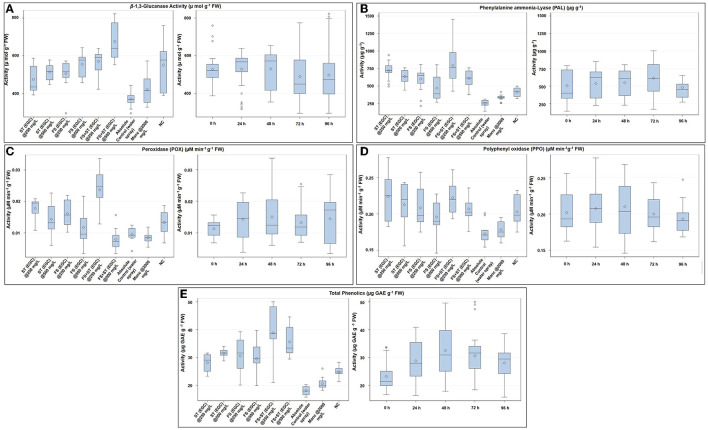
Box plot graphic representations of the effect of different treatments on enzymatic antioxidants viz., β-1, 3-glucanase **(A)**, phenylalanine ammonia-lyase **(B)**, peroxidase **(C)**, polyphenol oxidase **(D)**, and non-enzymatic antioxidant (total phenols) **(E)** in maize leaves. Horizontal lines within each box denote the median value (diamond within each box represents the average value), the lower and upper edges of the box represent the 25th and 75th percentile, whiskers denote the upper and lower ranges, and dots outside the box represent outliers; EGC, essential oil-grafted CuNPs; CuNPs, copper nanoparticles; EO, essential oil (clove oil); Manc, mancozeb 75% WP; ST, seed treatment; FS, foliar spray; negative control, only pathogen inoculate.

The β-1, 3-glucanase enzyme showed an increase in activity till 48 h, and then, it decreased over the next two time points (Figure 8A; Supplementary Table 7). ST+FS (EGC) at 500 mg L^−1^ exhibited a significant increase in the activity of 45.250% (*p* ≤ 0.05) as compared to the control (Figure 8A; Supplementary Table 7). Overall, the foliar spray combined with seed treatment of EGC resulted in the highest activity followed by foliar spray and seed treatment. Unexpectedly, Mancozeb at 2,000 mg L^−1^ also rendered an increase of 12.014% as compared to the control. The negative control also exhibited an increase, however, statistically, it was at par with the other treatments namely ST (EGC) at 500, FS (EGC) at 250 and 500, and ST+FS (EGC) at 250 mg L^−1^ (*p* ≤ 0.05) (Figure 8A; Supplementary Table 7).

The PAL enzyme did not follow a definite trend over time; however, an increase in the activity at 72 h and an abrupt decrease thereafter was observed (Figure 8B; Supplementary Table 8). The highest PAL activity was observed with ST+FS (EGC) at 250 mg L^−1^, significantly higher by 67.862% as compared to the control (*p* ≤ 0.05) (Figure 8B; Supplementary Table 8). At 0 h, ST (EGC) at 250 and 500 mg L^−1^ resulted in significantly high activity, which decreased thereafter (Figure 8B). As compared to the control, mancozeb at 2,000 mg L^−1^ had an increment of 22.883% (Supplementary Table 8). In the negative control, the increase in the activity was apparently high as compared to the control; nevertheless, the increase was significantly less as compared to the EGC treatments.

The POX enzyme activity was seen increasing up to 48 h and then followed a decreasing trend (Figure 8C; Supplementary Table 9). Maize plants treated with ST+FS (EGC) at 250 mg L^−1^ resulted in a significantly high activity of 59.447% (*p* ≤ 0.05) as compared to the control (0.010) and other treatments (24.929–64.241%) (Figure 8C; Supplementary Table 9). ST+FS (EGC) at 500 mg L^−1^ resulted in a drastic decrease in the POX activity over time, and the mean decrease was 18.081% compared to the control. It was observed that the lower concentration of 250 mg L^−1^, either ST or FS or ST+ST, rendered higher activity (Figure 8C). An increase in activity in the negative control was comparatively low as compared to the EGC treatments.

Similar to the β-1, 3-glucanase enzyme, a definite trend in the increase over time was observed in the case of PPO, which increased till 48 h and then decreased thereafter (Figure 8D; Supplementary Table 10). The highest PPO activity was observed with ST (EGC) at 250 mg L^−1^ with an increase of 22.939% over control (Figure 8D; Supplementary Table 10). However, other treatments of EGC were at par with ST (EGC) at 250 mg L^−1^, except for FS (EGC) at 500 mg L^−1^, which was at par with the control. The activity in the negative control was also at par with EGC treatments (Supplementary Table 10).

#### 3.5.2. Non-enzymatic antioxidants

Total phenolics in terms of μg GAE g^−1^ FW were significantly high in maize leaves treated with ST+FS (EGC) at 250 mg L^−1^, by an increase of 53.248% as compared to the control (*p* ≤ 0.05) (Figure 8E; Supplementary Table 11). Overall, the content increased up to 48 h and then declined eventually (Supplementary Table 11). In general, the total phenolic content in the EGC-treated maize leaves was significantly higher than the negative control (*p* ≤ 0.05) (Figure 8E; Supplementary Table 11).

### 3.6. Phytotoxic studies

#### 3.6.1. Physiological parameters

**Figure 9:**
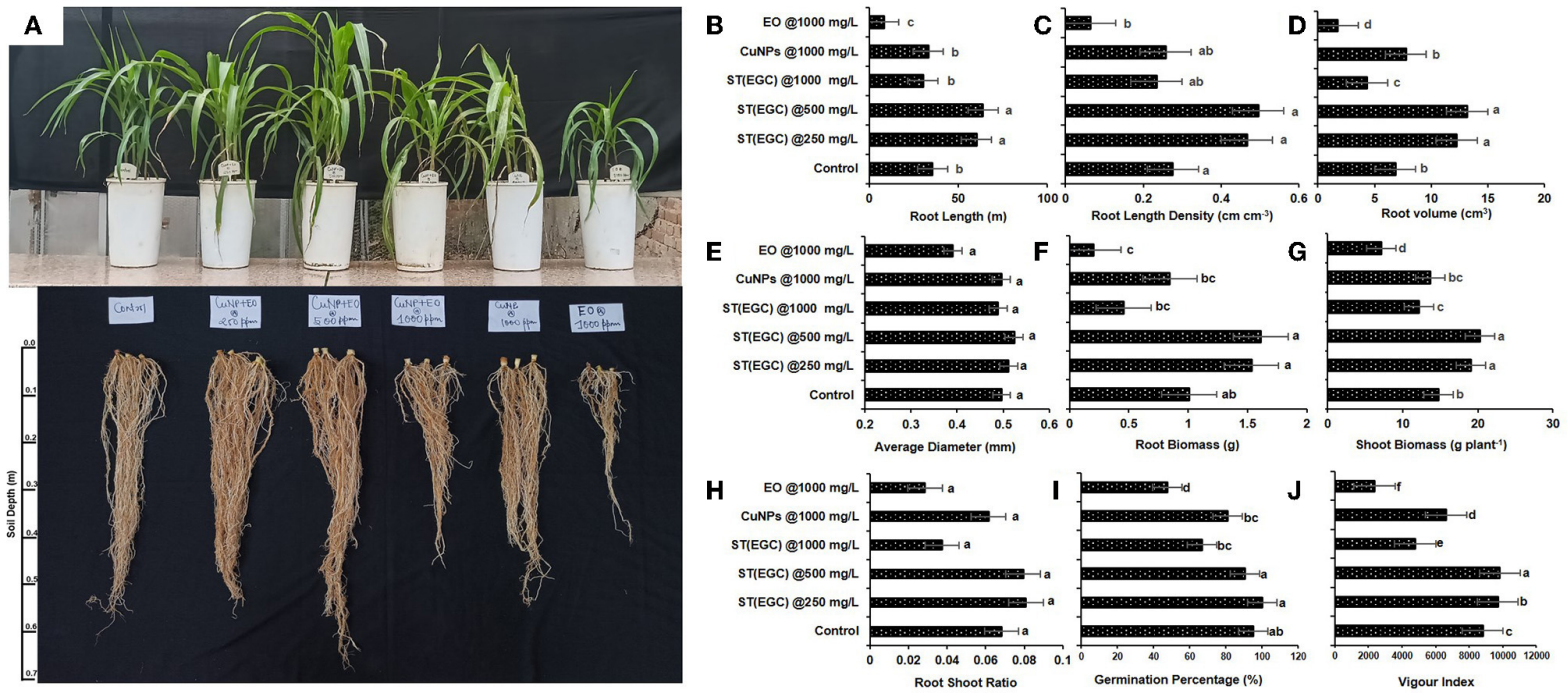
Visible effect of different treatments on shoot and root of maize **(A)** and concentration-dependent effect of different treatments on root length **(B)**, root length density **(C)**, root volume **(D)**, average diameter **(E)**, root biomass **(F)**, shoot biomass **(G)**, root–shoot ratio **(H)**, germination percentage **(I)**, and vigor index **(J)** exposed to EO-grafted CuNPs and grown in a pot. Data (mean ± standard errors) on the y-axis with different letters are significantly different in each graph (Tukey HSD, *p* ≤ 0.05). Data documented 35 DAS. Treatment used for 1st, 2nd, 3rd, 4th, 5th, and 6th pots are ST (EGC) @250 mg L^−1^, ST (EGC) @500 mg L^−1^, ST (EGC)@ 1,000 mg L^−1^, ST (CuNPs)@ 1,000 mg L^−1^, and ST (EO) @ 1,000 mg L^−1^, respectively. ST, seed treatment; EGC, essential oil-grafted CuNPs; CuNPs, copper nanoparticles; EO, essential (clove oil).

A pot experiment was conducted to determine the effect of EGC on different physiological parameters (Figure 9A). Germination percentage was significantly reduced due to EO at 1,000 mg L^−1^ (47.619%) followed by ST (EGC) at 1,000 mg L^−1^ (66.667%) and CuNPs at 1,000 mg L^−1^ (80.952%) (*p* ≤ 0.05). Among the treatments, ST (EGC) at 250 mg L^−1^ resulted in 100% germination which was significantly higher than the control (95.238%) (*p* ≤ 0.05) (Figure 9I; Supplementary Table 12).

Vigor index was perceived as significantly high in ST (EGC) at 500 mg L^−1^ (*p* ≤ 0.05). ST (EGC) at 250 mg L^−1^ also resulted in a significantly high vigor index (9,690.223) as compared to the control (8,799.206) (*p* ≤ 0.05). The least significant vigor index (2,366.984) was observed in plants treated with ST (EO) at 1,000 mg L^−1^. ST (EGC) at 1,000 mg L^−1^ also caused a reduction in the vigor index (4,787.143) (Figure 9J; Supplementary Table 12).

WinRHIZO professional software (LA2400, Regent instrument, Quebec, Canada) was used to determine the effect of treatments on root length, root length density, root volume, and average diameter of the roots (Supplementary Figure 4). ST (EGC) at 500 mg L^−1^ resulted in a significant increase in root length (63.896 m), root length density (0.495 cm cm^−1^), root volume (13.167 cm^3^), and average diameter (0.524 mm) of the roots (*p* ≤ 0.05) (Figures 9B–E; Supplementary Table 12). ST (EGC) at 250 mg L^−1^ also led to a significant increase in all the root parameters under study as compared to the control; however, the increase was insignificant in concern to the average diameter. The treatment ST (EO) at 1,000 mg L^−1^ rendered a significant decrease in all the root parameters under study *viz.*, root length, root length density, root volume, and average diameter by 15.240, 15.240, 36.824, and 1.576%, respectively.

Dry shoot and root biomass per plant were found significantly the highest in the ST (EGC) at 500 mg L^−1^, a percentage increase of 27.217 and 37.418%, respectively (*p* ≤ 0.05). ST (EGC) at 250 mg L^−1^ rendered an increase in biomass of shoot and root by 22.684 and 34.301%, respectively, as compared to the control (Figures 9F, G; Supplementary Table 12). However, higher concentrations of ST (EGC) at 1,000 mg L^−1^ led to a reduction in the biomass of shoot and root by 17.535 and 54.749%, respectively. Furthermore, ST (EO) at 1,000 mg L^−1^ resulted in a significant reduction in the biomass of shoot and root by 51.862 and 79.834%, respectively (*p* ≤ 0.05). Regarding the root–shoot ratio, ST (EGC) at 250 mg L^−1^ and ST (EGC) at 500 mg L^−1^ rendered an increase of 15.657 and 14.165%, respectively, as compared to the control (Figure 9H; Supplementary Table 12). On the contrary, ST (EO) resulted in a drastic decrease in a root–shoot ratio by 58.196% followed by (EGC) at 1,000 mg L^−1^ and ST (CuNPs) at 1,000 mg L^−1^, a decrease of 45.164 and 9.774%, respectively.

#### 3.6.2. Histological studies

**Figure 10:**
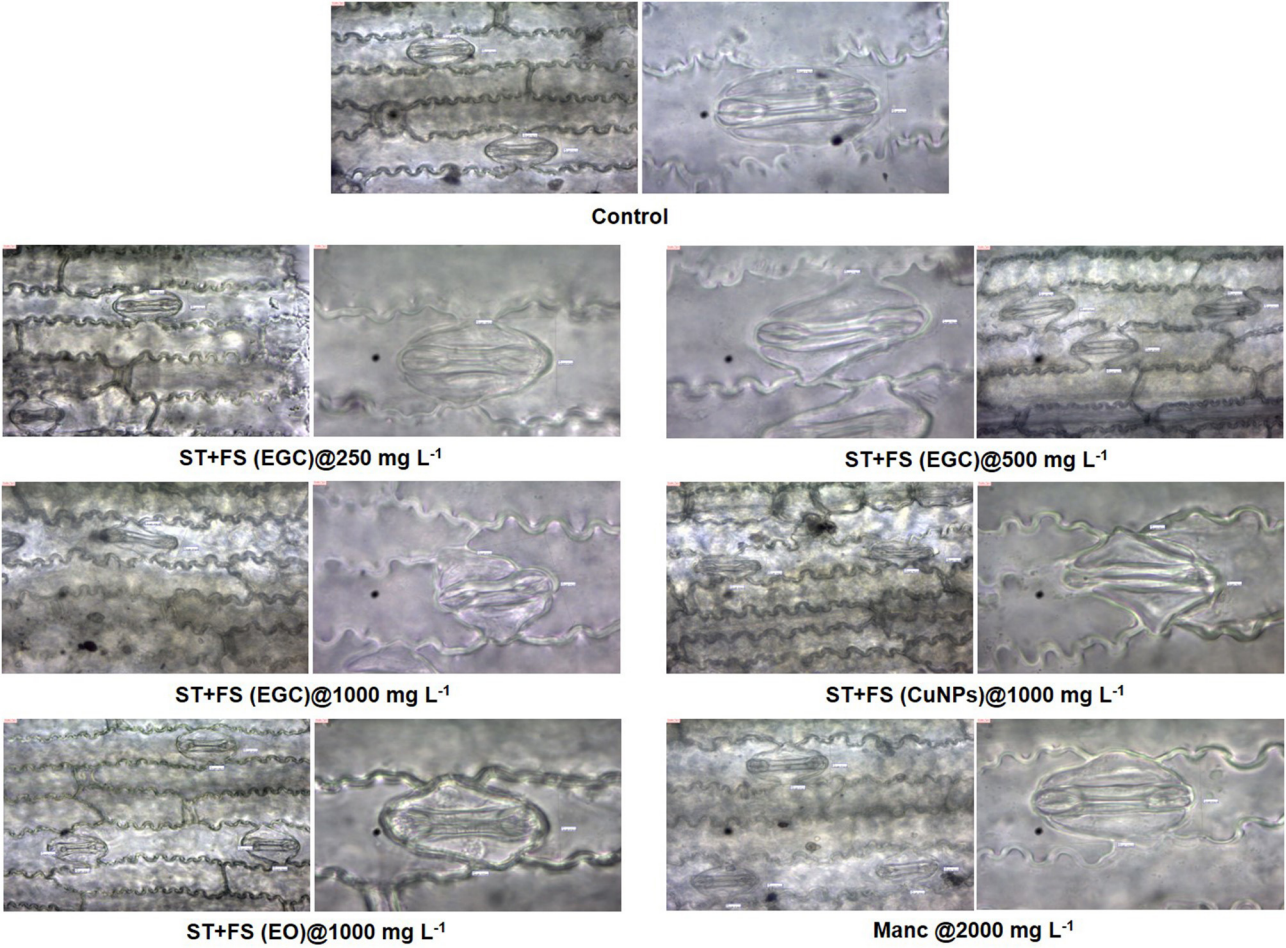
Effect of different treatments on guard cells of stomata of maize 2 days after spray which were seed-treated before sowing, observed under light microscope at 40X and 100X. FS, foliar spray; ST, seed treatment; EGC, essential oil-grafted CuNPs; CuNPs, copper nanoparticles; EO, essential oil (clove oil).

Histological studies of leave tissues, in particular, the stomata and its guard cell, provided a better insight into the effect of EGC, CuNPs, EO, and mancozeb on the maize plant. Microscopy revealed a slight and high distortion/damage to guard cells at the concentration of 500 and 1,000 mg L^−1^, respectively, due to EGC used as a seed treatment+foliar spray (Figure 10). Moreover, FS+ST of EGC at 1,000 mg L^−1^ resulted in a considerable reduction in the size of guard cells to 81 × 62 μm compared to the control (116 × 64 μm). FS+ST (EGC) at 250 mgL^−1^ did not cause any distortion; also, shape and size remained the same as the control, i.e., 116 × 64 μm. FS+ST of CuNPs at 1,000 mg L^−1^ also damaged the guard cells to a large extent and reduced the size of guard cells to 96 × 72 μm. No significant damage or shrinkage of guard cells was observed in the case of FS+ST treatments of EO and mancozeb 75% WP at the rate of 1,000 and 2,000 mgL^−1^, respectively (Figure 10).

#### 3.6.3. Photosynthetic pigments

**Figure 11:**
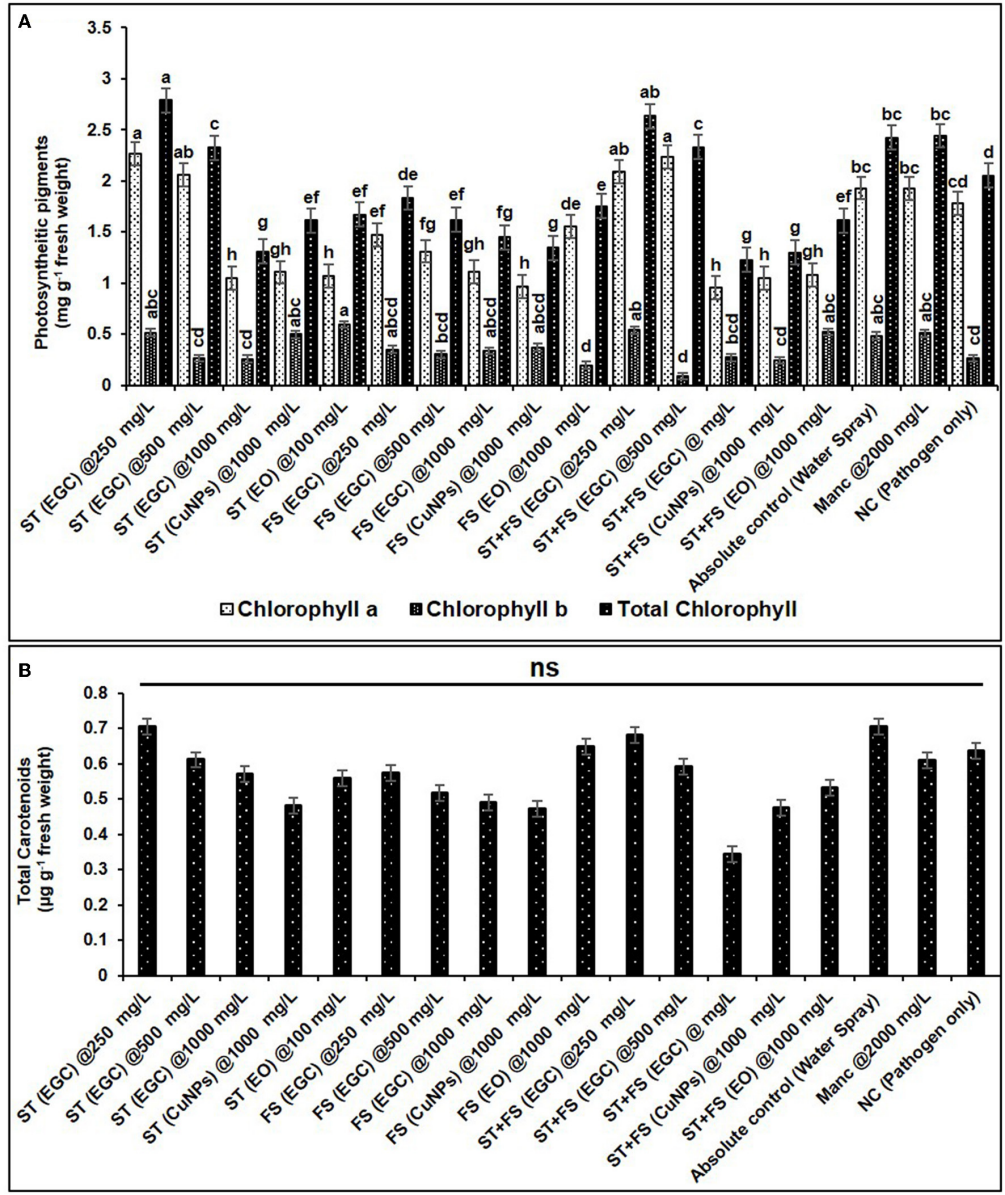
Effect of different treatments and their concentrations on photosynthetic pigments viz., chlorophyll a, b, and total chlorophyll **(A)** and total carotenoids **(B)**. The data are the mean of four replications. Data (mean ± standard errors) with different letters are significantly different in each graph (Tukey HSD, *p* ≤ 0.05), and “ns” represents no significant difference between the treatments.

Photosynthetic pigments are important indicators of stress (MacFarlane and Burchett, 2001; Chaves et al., 2009). The chlorophyll “*a*” (*Chl a*) significantly increased in maize plants treated with ST (EGC) at 250 mg L^−1^ (2.264 mg g^−1^ FW) and ST+FS (EGC) at 500 mg L^−1^ (2.232 mg g^−1^ FW) as compared to the control (1.930 mg g^−1^ FW) (*p* ≤ 0.05) (Figure 11A, Supplementary Table 13). However, a higher concentration of 1,000 mg L^−1^ of EGC, CuNPs, and EO resulted in a significant reduction in *Chl a* content, ST+FS (EGC) at 1,000 mg L^−1^ causing the extreme reduction of 50.722% compared to control. As expected, in the negative control (plant inoculated with pathogen only), the content was less, however, statistically (*p* ≤ 0.05) at par with the control. In the case of chlorophyll ‘***b***' (*Chl b*), ST (EGC), and ST+FS (EGC) at 250 mg L^−1^ led to an increase of 5.729 and 10.0119%, respectively (Figure 11A, Supplementary Table 13). In addition, ST (CuNPs) at 1,000 mg L^−1^ resulted in the increase in *Chl b* content by 2.589%. Intriguingly, ST (EO) at 1,000 mg L^−1^ and ST+FS (EO) at 1,000 mg L^−1^ also rendered an increase in *Chl b* content. The rest of the treatments rendered a decrease in *Chl b* content, ST+FS (EGC) at 1,000 mg L^−1^ causing the highest decrease of 81.456%. Total chlorophyll content was found increased in the treatments *viz.*, ST (EGC) at 250 mg L^−1^ (2.790 mg g^−1^ FW) and ST+FS at 250 mg L^−1^ (2.636 mg g^−1^ FW) by 13.059 and 7.991%, respectively, where the former being significantly high as compared to the control (*p* ≤ 0.05) (Figure 11A, Supplementary Table 13). Other treatments under study resulted in a significant decrease in total Chlorophyll, and the decrease due to ST+FS (EGC) at 1,000 mg L^−1^ was the highest. Concerning mancozeb 75% WP treatment at 2,000 mg L^−1^, an insignificant increase of 4.514 and 0.641% was perceived in *Chl b* and total chlorophyll content, respectively, and a decrease of 0.386% in *Chl a*.

No significant change was observed in total carotenoids due to the treatments. However, a relatively higher carotenoid content of 0.706 μg g^−1^ FW was discerned in the plant treated with ST (EGC) at 250 mg L^−1^. The lowest total carotenoid content was found in the plant treated with ST+FS (EGC) at 1,000 mgL^−1^ (0.345 μg g^−1^ FW) (Figure 11B, Supplementary Table 13).

#### 3.6.4. Bioaccumulation of copper in maize leaves and its effect on zinc and manganese

The copper concentrations in maize leaves treated with ST+FS (CuNPs), ST+FS (EGC), and FS (EGC) FS (CuNPs) at 1,000 mg L^−1^ were estimated as high as 399.950, 375.795, 329.430, and 306.430 mg kg^−1^ DW, respectively, which were significantly higher as compared to control (10.570 mg kg^−1^) (*p* < 0.05) (Table 4). The Cu concentration in the maize leaves with seed treatment at different concentrations, i.e., 250, 500, and 1,000 mg L^−1^ of EGC, was found to be 21.210, 25.690, and 65.110 mg kg^−1^ and 1,000 mg L^−1^ of CuNPs resulted in 64.571 mg kg^−1^ which were also significant as compared to the control (*p* < 0.05). However, the Cu concentration in the EO-treated plant was at par with the control, and mancozeb-treated plant also had not had much effect on the Cu concentration (Table 4). The concentration of Cu had a negative correlation with two other elements under study, *viz.*, Mn and Zn. In both cases, the concentration was found to be significantly lower (*p* < 0.05) in the maize leaves treated with a higher concentration of EGC, which was low as 3.187 mg kg^−1^ in ST (EGC) at 500 mg L^−1^ for Mn and as low as 8.575 mg kg^−1^ in ST+FS (EGC) at 500 mg L^−1^ for Zn (Table 4).

**Table 4:** Bioaccumulation of copper in maize leaves under different treatments and its effect on manganese and zinc concentration.

**S. No**	**Treatment**	Cu concentration (mg kg^−1^ Dry weight)^*^	Mn concentration (mg kg^−1^ Dry weight)^*^	Zn concentration (mg kg^−1^ Dry weight)^*^
1	ST (EGC) @250 mg L^−1^	21.210 ± 0.593^k^	6.407 ± 0.546^de^	12.390 ± 0.162^ghi^
2	ST (EGC) @500 mg L^−1^	25.690 ± 0.421^j^	6.687 ± 0.748^de^	11.970 ± 0.359^hi^
3	ST (EGC) @1,000 mg L^−1^	65.110 ± 0.404^i^	3.187 ± 0.141^g^	9.850 ± 0.203^jkl^
4	ST (CuNPs) @1,000 mg L^−1^	64.517 ± 0.619^i^	5.953 ± 0.290^e^	9.625 ± 0.169^kl^
5	ST (EO) @100 mg L^−1^	9.413 ± 0.465^l^	7.280 ± 0.398^de^	15.225 ± 0.398^d^
6	FS (EGC) @250 mg L^−1^	76.687 ± 2.067^h^	9.973 ± 0.458^bc^	14.595 ± 0.571^de^
7	FS (EGC) @500 mg L^−1^	140.777 ± 0.566^f^	6.440 ± 0.081^de^	12.635 ± 0.613^fgh^
8	FS (EGC) @1,000 mg L^−1^	329.827 ± 4.099^c^	5.543 ± 0.242^ef^	10.885 ± 0.429^ijk^
9	FS (CuNPs) @1,000 mg L^−1^	306.430 ± 1.961^d^	8.217 ± 0.217^cd^	13.580 ± 0.199^efg^
10	FS (EO) @1,000 mg L^−1^	9.060 ± 0.323^l^	12.493 ± 0.604^a^	14.093 ± 0.088^def^
11	ST+FS (EGC) @250 mg L^−1^	97.380 ± 1.961^g^	6.965 ± 0.273^de^	11.270 ± 0.076^hij^
12	ST+FS (EGC) @500 mg L^−1^	180.640 ± 0.374^e^	3.815 ± 0.264^fg^	8.575 ± 0.187^l^
13	ST+FS (EGC) @1,000 mg L^−1^	375.795 ± 0.177^b^	3.780 ± 0.210^fg^	8.645 ± 0.193^l^
14	ST+FS (CuNPs) @1,000 mg L^−1^	399.950 ± 1.957^a^	3.500 ± 0.150^g^	14.595 ± 0.107^de^
15	ST+FS (EO) @1,000 mg L^−1^	8.717 ± 0.061^l^	9.663 ± 0.291^bc^	14.595 ± 0.107^de^
16	Absolute control (Water Spray)	10.570 ± 0.205^l^	13.300 ± 0.153^a^	23.975 ± 0.158^a^
17	Manc @2,000 mg L^−1^	10.957 ± 0.205^l^	10.535 ± 0.247^b^	17.500 ± 0.203^c^
18	NC (Pathogen only)	11.353 ± 0.267^l^	12.600 ± 0.351^a^	19.425 ± 0.122^b^

^*^Data are the mean of two different seasons with three replications. Data (mean ± standard errors) followed by different letters in each column indicate a significant difference (Tukey HSD, *p* ≤ 0.05). ST, seed treatment; FS, foliar spray; EGC, EO-grafted CuNPs; EO, essential oil (clove oil); Manc, mancozeb 75% WP; NC, negative control.

## 4. Discussion

Sustainable agriculture is crucial considering the population explosion, and to support sustainable agriculture, nanoformulations with high efficacy and considerably benign to the environment are produced in the recent past (Fraceto et al., 2016). The main objective of employing nanoparticles in plant protection was to attain a higher efficacy while at the same time reducing pesticide usage to have an environmentally friendly approach. Apart from the excellent antibacterial and antifungal activity, several nanoparticles such as CuNPs are capable of inducing a cascade of defense biochemical and gene responses allowing the plant to mitigate biotic stress (Divya et al., 2020; Kongala et al., 2021). In the recent past, clove oil has been documented to have strong antifungal and antimicrobial activity against a wide range of pathogens (Pinto et al., 2009; Muñoz Castellanos et al., 2020; Purkait et al., 2020).

The characterization is performed to confirm the formation of NPs and to determine the shape, size distribution, surface chemistry, particle charge, and stability (Mourdikoudis et al., 2018). In our study, with the addition of reducing agents and advancement in time, the color gradually changed from blue to yellow-green and to reddish brown indicating a size reduction. Similar observations have been made by Dang et al. (2011), Umer et al. (2014), Khalid et al. (2015), and Markin and Markina (2019) where the final solution of CuNPs assumed reddish brown color. The characteristic absorption band (λ_max_) for the CuNPs lies between 500 and 600 nm (Rahimi et al., 2010; Dang et al., 2011; Ramyadevi et al., 2011). We observed the characteristic peak at 566 nm when EGC was subjected to UV-vis spectroscopy. The characteristic peak revealed in the UV-Vis absorption spectrum is due to the surface plasmon resonance (SPR) of the synthesized CuNPs (Swarnkar et al., 2011; Jhuang et al., 2021). In concurrence with our work, Rahimi et al. (2010), Dang et al. (2011), Aissa et al. (2015), and Seku et al. (2018) reported plasmon resonance peaks at 558 nm, 576 nm, 562 nm, and 570 nm, respectively, for CuNPs. TEM measured spherical CuNPs with an average size of 30.54 nm. However, EGC was observed to be agglomerated and the average particle size was also increased to 38.48 nm, which could be due to the adsorption of EO (clove oil) around the CuNPs. An agglomeration to a certain extent was also observed in EGC. XRD data revealed that diffraction peaks were in accordance with standard JCPDS database No. 040836 for CuNPs, confirming the face-centered cubic phase of CuNPs in EGC. Our result is in tune with the work reported by Umer et al. (2014), Khan et al. (2016), Dong et al. (2018), and Phul et al. (2018) where they also observed similar diffraction peaks. DLS and zeta potential techniques provide valuable information about the size distribution and surface charge of nanoparticles indicating their stability and aggregation behavior. In our investigation, we obtained a hydrodynamic particle size of 91.28 nm which is greater than the average size measured using TEM and XRD. The reason for the relatively larger size in DLS is the hydrodynamic state of the nanoparticle, i.e., the presence of H_2_O around the particles (Khlebtsov and Khlebtsov, 2011). The polydispersity index of 1 indicates the particles are monodispersed. The zeta potential of particles of −21.5 mV indicated a relatively stable suspension of CuNPs. The higher charge results in higher stability. The negative charge on the surface can be linked to the hydroxyl groups that form as particles are dispersed in water solvent (Anandhavalli et al., 2015) and may be due to the presence of the OH group of eugenol present in the formulation. Similar to our result, Kruk et al. (2015) recorded a negative zeta potential of −40 mV and an average particle size of 50 nm. In a similar investigation, Chandraker et al. (2020) obtained an average particle size of 80 nm. Manikandan and Sathiyabama (2015) also recorded chitosan-CuNPs of 88.21 nm and negative zeta potential of −29.0 mV and DLS revealed larger particle size than TEM (20–30 nm). FTIR analysis identifies functional groups, polymeric, organic, and inorganic compounds associated with the compound of interest by measuring the infrared intensity vs. wavelength of light. FTIR spectra showed significant peaks at 3,737 cm^−1^ for hydroxyl groups of alcohol and a broad peak at 2,918 cm^−1^ for the presence of secondary amines in clove oil (Eugenol). Two peaks at 1,417 and 1,619 cm^−1^ correspond to the –CH bending of alkanes and –CO stretching, respectively. This is in agreement with previous study reported by Rajesh et al. (2017). Furthermore, IR spectra showed two peaks at 1,684 and 1,753 cm^−1^ corresponding to CuNPs (Dang et al., 2011). Ester bond was located at wave number 1,095 cm^−1^ in CuNPs (Dang et al., 2011). As per the IR spectra, B-H stretching was located at 2,323 and 2,011 cm^−1^ corresponding to NaBH_4_ (D'Anna et al., 2014). The IR spectra also revealed a shift of peaks at 3,021, 1,516, and 1,355 cm^−1^ representing hydroxyl band, acidic asymmetric stretching, and –CH deformations, respectively, in ascorbic acid (Umer et al., 2014). GC-MS analysis demonstrated minimum deterioration of EO when combined with CuNPs. There are several reports which advocate the increased efficacy of essential oil when loaded with NPs due to better penetration and increased affinity for targets (Bazana et al., 2019; Ghodrati et al., 2019; Luis et al., 2020).

The EGC exhibited significant antifungal activity at 20 μg ml^−1^ and complete inhibition at 100 μg ml^−1^. A synergistic effect was discerned against five different maize pathogens under the study. In all the cases, EGC performed significantly better than the commercial fungicides at the same concentrations (100 μg ml^−1^). In addition, in our study, the ED_50_ was achieved between 46 and 56 μg ml^−1^, a concentration of compound required to arrest or inhibit the biological activity by 50%, thus giving an idea about the antagonistic efficacy of fungicides (Miller and Tainter, 1944). However, antifungal activity varies and depends on the types of pathogens it was used against. Similar to the present study, an excellent antifungal activity of thyme and dill essential oil encapsulated in CuNPs against *Colletotrichum nymphaeae* with 90% inhibition was reported by Weisany et al. (2019). In addition, chitosan-based nanocomposite loaded with essential oil (thyme-oregano, thyme tea tree, and thyme peppermint) was demonstrated to reduce the growth of post-harvest fungi *viz., Aspergillus flavus, A. niger, A. parasiticus*, and *Penicillium chrysogenum* by 51–77% (Hossain et al., 2019). The synergistic antimicrobial effect of a combination of essential oil and NPs has been reported against several pathogens, for instance, silver nanoparticles (AgNPs) in combination with thymol (Taghizadeh and Solgi, 2014), silver nano functionalized with *Cymbopogon citratus, Eucalyptus globules, Azadirachta indica*, and *Ocimum sanctum* essential oil (Bansod et al., 2015), a combination of mesoporous silica NP with cinnamon and clove essential oil as nanoencapsulation against various pathogens such as *Candida albicans, Staphylococcus aureus, Pseudomonas aeruginosa*, and *Escherichia coli* (Lillie, 2016).

The CuNPs have remarkable antifungal activity. Pariona et al. (2019) reported the antifungal activity of CuNPs against *Fusarium solani, Neofusicoccum* sp., and *Fusarium oxysporum* causing 55, 95, and 97% inhibition of radial growth at 0.1, 0.5, and 0.5 mg ml^−1^, respectively. Similarly, Kamel et al. (2022) reported Cu_2_O NPs of 100 μg ml^−1^ could render inhibition percentage to 78.89%. Unlike, the present study's result, Banik and Pérez-de-Luque (2017) reported a high CuNPs concentration (100 and 800 mg L^−1^) to be effective against *Phytophthora cinnamon* and *Alternaria alternata*. Similarly, a high concentration of CuNPs (450 mg L^−1^) was used to achieve significant inhibition against *Fusarium* sp. (Viet et al., 2016). As compared to aforesaid reports, in our study, at much lower concentrations (below 100 μg ml^−1^), better efficacy was observed confirming the contribution of EO toward the antifungal activity. The low polydispersity and extremely small size and high surface-to-volume ratio of CuNPs in EGC are another plausible reason for the enhanced efficacy. CuNPs oxidize and release cupric ions (Cu^2+^ and Cu^+^) resulting in toxic hydroxyl-free radicals which degenerate the membrane integrity, damage the enzymes by binding to the sulfhydryl amino and carboxyl groups of amino acids, and disrupt the biochemical processes and the DNA helix of the target pathogens leading to cell death (Wei L. et al., 2010; Wei Y. et al., 2010; Ramyadevi et al., 2012). The mechanism by which essential oil inhibits the pathogens can be attributed to the functional groups present and their interaction with other components (Dorman and Deans, 2000). Essential oil used under the current investigation has eugenol as the main component which has phenol as a functional group containing a hydroxyl, contributing toward its activity. It has been understood that the relative position of hydroxyl is imperative for the activity (Veldhuizen et al., 2006). The eugenol causes damage and disruption, hyperpolarization to the cell membrane increasing the membrane permeability and leading to leakage of ions and important cell contents, eventually rendering cell death (Ulanowska and Olas, 2021).

Maydis leaf blight (MLB) disease caused by *Bipolaris maydis* is a dreadful foliar maize disease prevalent in the Indian plains, hills, and peninsular regions (Kumar et al., 2016). *In vivo* evaluation of EGC revealed the better performance of seed treatments than spray treatments, which can be attributed to the activation of defense genes in the maize at an early stage and can be assumed, the role of EGC in priming. However, the combination of seed treatment and foliar spray was remarkable indicating the prophylactic and curative effects of EGC. Among the different treatments, ST+FS (EGC) at 1,000 mg L^−1^ resulted in the least PDI, but germination and growth were highly compromised and leaf chlorosis was also visible on the plants. ST+FS (EGC) at 500 mg L^−1^ was considered the best treatments as PDI and phytotoxicity were considerably less and performed significantly better than commercial fungicide. EO alone evinced high PDI, highest in FS (EO) at 1,000 mg L^−1^. Perhaps, the volatility of EO in an open atmosphere led to its rapid loss before it could act on the pathogen. Nevertheless, the synergistic effect of EO and CuNPs when used in combination was well discerned in the study. In the previous study, it was observed that CuNPs could substantially reduce the PDI of diseases namely BLSB (*R. solani* f. sp. *sasakii*) and MLB (*B. maydis*) when applied as seed treatment supplemented with spray at 300 mg L^−1^ (Dorjee et al., 2023). Similar to the current study, Lamsal et al. (2011) demonstrated a reduction in PDI of powdery mildew to 20% at 100 ppm of AgNPs spray as compared to the control 82% in cucumber plant and pumpkin in *in vivo* condition. The efficacy of the EGC in the field condition is conceivably due to the direct effect on the pathogen's membrane and disrupting physiology. The induction of antioxidant defense gene and other biochemical responses (Sathiyabama and Manikandan, 2016; Choudhary et al., 2017; Zhao et al., 2017) against the pathogens due to the oxidative stress of CuNPs is another plausible reason for its excellent efficacy, thus proving EGC as prophylactic and curative.

We investigated biochemical responses in maize leaves under the influence of EGC. Except for β-1, 3-glucanase, a seed treatment, foliar spray, or a combination of foliar spray and seed treatment at 500 mg L^−1^ caused an insignificant increase, indicating high toxicity at higher concentrations that the maize plant could not overcome. CuNPs being metallic in nature create oxidative stress by generating reactive oxygen species (ROS), and to detoxify, enzymatic and non-enzymatic antioxidants are produced by the plants which further leads to speculated defense mechanisms. In addition, an essential oil such as eugenol (Zhu et al., 2022), tea tree oil (Shao et al., 2013), and thyme oil (Perumal et al., 2017) has been found to enhance the antioxidant enzymatic activities including POX chitinase, PAL, β-1,3-glucanase, catalase, and SOD against oxidative stress.

POX plays a key role in plant defense by cross-linking cell wall components, phytoalexin synthesis, and lignin–suberin formation (Almagro et al., 2009) and induced by biotic agents (Rodriguez and Flores, 1987; Choi et al., 2007; Souza et al., 2010; Lanubile et al., 2015) and abiotic agents (Sasaki et al., 2007). We recorded a significant increase in POX activity of 59.447% in pre-inoculated maize leaves treated with 250 mg L^−1^ of EGC. In agreement with our result, an increase in POX activity due to CuNPs treatment was observed by Di et al. (2023) in *Brassica Chinensis* L.; however, they did not find any significant increase due to CuO NPs. Similarly, Hayat et al. (2021) also reported a significantly high activity of POX upon CuNPs treatment in *Artemesia absinthium* L. In concurrence with our result, Khan et al. (2020) reported the induction of POX in *Vicia faba* L. using 15 mg L^−1^ nano-titanium dioxide. Intriguingly, Jurkow et al. (2020) reported that Au-NPs increased the total peroxidase activity; however, AgNPs and Pt-NPs decreased the activity.

PPOs are induced by biotic and abiotic stress and can oxidize catechol and caffeic acid to quinones, which are highly reactive and toxic with a definite role in plant defense (Constabel and Barbehenn, 2008; Zhang and Sun, 2021). In our study, PPO activity was more in seed-treated maize than in spray-treated, which could be the result of better internalization of Cu due to the longer time of exposure, thereby increasing the copper-containing enzyme PPO. A similar effect of CuNPs on PPO activity was reported by Essa et al. (2021), where they observed that higher concentration led to an increase in the activity of PPO and a decrease in the activity of other enzymes like POX and CAT.

β-1, 3-glucanases have broad-spectrum antifungal activity and play an important role in plant developmental processes (Perrot et al., 2022). We observed a higher increase in β-1, 3-glucanase activity in maize leaves inoculated with *B. maydis* of 675.500 μmol g^−1^ FW, when exposed to EGC compared to maize treated with *B. maydis* alone (550.667 μmol g^−1^ FW). In concurrence with our findings, Sarkar et al. (2020) reported a 2.04-fold change of β-1, 3-glucanases activity in *Lens culinaris* roots treated with 0.05 mg mL^−1^ CuO NPs. Similarly, an increase of 29.26% in activity due to chitosan NPs was observed by Chandra et al. (2015).

PAL is involved in the phenylpropanoid pathway and biosynthesis of polyphenol and benzoic acid (Lee et al., 1995), and the latter is responsible for the synthesis of salicylic acid (SA). SA plays a vital role in defense signaling, initiating systemic resistance, and defending plants against pathogens (Rivas-San Vicente and Plasencia, 2011). An increase in PAL activity was well pronounced in the present study, suggesting that EGC could activate SA-mediated defense response (Dempsey et al., 2011). Consistent with our result, an increase in PAL activity due to CuNPs treatment has been reported by Quiterio-Gutiérrez et al. (2019) and Hernández-Hernández et al. (2019) in tomato, Zare et al. (2020) in Purslane leaves, and Lala (2020) in *Bacopa monnieri* (L.). The increase in PAL could also be attributed to the eugenol present in EGC as well due to its ability to induce PAL activity (Zhu et al., 2022).

We investigated the change in total phenolics due to EGC, a non-enzymatic antioxidant with a vital role in defense. Phenolics are secondary metabolites with a benzene ring and one or more than one hydroxyl group, which plays an imperative role in plant defense by acting as phytoanticipins and phytoalexins against pathogens (Lattanzio et al., 2006). In agreement with previous reports by Quiterio-Gutiérrez et al. (2019) and Sarkar et al. (2022), where they found enhanced total phenolics due to Se/Cu NPs and Chitosan NPs in tomato and chili, respectively, against *Alternaria* sp., we also observed an increase in the total phenol due to EGC treatments in maize leaves. In agreement with our report, an increase in total phenol due to CuNPs (50 mg kg^−1^) in wheat was reported by Noman et al. (2020). A similar result has been demonstrated by Lala (2020) for an increase in phenolic content of 116% in *Bacopa monnieri* when treated with CuNP concentration of 75 mg L^−1^. Similar to enzymatic antioxidants studied, seed treatment in combination with spray rendered a higher increase in total phenolics as well. Moreover, silver nanoparticles (Chung et al., 2017), nano-titanium dioxide (Ghorbanpour, 2015), AgNPs and Pt-NPs (Jurkow et al., 2020), and ZnO (El-Zohri et al., 2021) also led to an accumulation of total phenolics in *Eclipta prostrata* leaves, *Salvia officinalis*, lettuce, and tomato, respectively. In addition to CuNPs, EO oil used must have also contributed either in enhancing or maintaining the phenolic content as reported by Perumal et al. (2017) in *Mangifera indica* L. using thyme oil.

At higher concentrations of 1,000 mg L^−1^ of EGC or CuNPs, phytotoxic symptoms on leaves were easily noticeable in the form of chlorosis, and microscopy revealed a reduction in size and damage to guard cells. In contrast, lower concentrations of EGC, i.e., 250 and 500 mg L^−1^, as a seed treatment resulted in higher chlorophyll and carotenoid content and negligible effect on guard cells, hence increasing the photosynthetic activity which in turn increased the biomass. We also observed a stimulating effect on various maize seedling characters. Cu plays a pivotal role in the electron transport chain, photophosphorylation, and cell wall metabolism and also enhances photosynthetic activity (Pradhan et al., 2015; Mir et al., 2021). Nevertheless, in our study, toxicity at higher concentrations can be attributed to the overabundance of Cu ions that created oxidative stress, interfering with the chloroplast, causing DNA damage and apoptosis, which led to decreased photosynthetic activity and also affecting plant height and stem diameter, germination, and eventually a reduction in the biomass (Marques et al., 2018; Gong et al., 2021). Excess Cu disrupts vital enzymes associated with the biosynthesis of chlorophyll by replacing Zn (Valasata et al., 2018) and alters the composition of protein affecting the photosynthetic membrane (Karimi et al., 2012). Cu stress rendering reduction in chlorophyll content and biomass has been studied in many plants and their species such as *Oryza Sativa* L. (Yang et al., 2020), lentil (Hossain et al., 2020), *Avicennia germinans* (González-Mendoza et al., 2013), *Brassica pekinensis* Rupr. (Xiong et al., 2006), *Origanum vulgare* subsp. *Hirtum* (Panou-Filotheou et al., 2001), and *Triticum aestivum* L. (Singh et al., 2007). Consistent with the current findings, a significant increase in photosynthesis and biomass of *Brassica* sp. by 10.2–19.6% was recorded when treated with CuO NPs and CuNPs at an optimum concentration (Wang et al., 2022). Similarly, Shende et al. (2017) also documented a remarkable promotion in *Cajanus cajan* L. growth when treated with optimum CuNPs concentration. Zakharova et al. (2019) also observed a positive effect of CuNPs (0.1 mg L^−1^) on wheat seed germination and seedling growth. A similar result in support of our study was also obtained by Adhikari et al. (2016), where they observed CuNPs at optimum lower concentrations improved germination and growth in maize, while higher concentrations proved to be detrimental. The increase in biomass and photosynthesis is correlated, and the data of the present study are in consensus. From data generated in the current investigation and earlier reports, it can be concluded that the negative or positive consequences of the nanoparticle are dose-dependent and may also vary from one crop species to another.

The phytotoxicity of essential oil limits its advances in bio formulation. In the pot experiment and in the field, it was observed that the seed treated with EO (Clove oil) exhibited significantly poor germination and poor growth indicating the contribution of EO in the phytotoxicity of EGC as well. Poor germination could be because of an increase in ABA content in treated maize seeds (Hu et al., 2017). Meyer et al. (2008) reported toxicity of clove oil against *Solanum lycopersicum, Capsicum annuum, Cucumis sativus*, and *Cucumis melo* at concentrations of 0.2 and 0.3% resulting in 0 to 50% seedling survival. In another study on weeds namely *Trifolium incarnatum* and *Vicia sativa*, a reduction in germination by 58.6 and 62.1%, respectively, was observed due to clove oil treatment (Vitalini et al., 2022). It has been established that eugenol acts as a major phytotoxic component of clove oil whereas β-caryophyllene and α-humulene had a minor role (Stokłosa et al., 2012).

Cu is required in trace quantity and the critical deficiency level fall in the range of 1–5 mg kg^−1^ DW, and the threshold limit for toxicity is over 20–30 mg kg^−1^ DW of the plant (Marschner, 1995; Nguyen et al., 2017). We observed that the bioaccumulation of Cu in seed treatment with EGC at 250 and 500 mg L^−1^ was 21.210 and 25.690 mg kg^−1^ DW, respectively, which is in the optimum range, although significantly high than the control. This indicates the translocation of Cu to aerial parts with growth. However, a higher concentration (spray or seed treatment and spray combined) led to an immoderate accumulation to a toxic level which could be due to residual unabsorbed Cu on leaves along with the bio-accumulated one. Thus, with the increase in dose, bioaccumulation also increased. In agreement with the current study, Zhao et al. (2017) also observed high bioaccumulation of Cu (523 mg kg^−1^) in maize leaves treated with CuO NPs at 100 mg L^−1^. Similarly, significant uptake of nano Cu compared to bulk Cu by 250% was also reported by Ogunkunle et al. (2018). In the current investigation, it was also observed that Cu concentration was negatively correlated with Mn and Zn homeostasis. Zinc is an important micronutrient and has a structural role in regulatory proteins (Berg and Shi, 1996), and for normal growth, an optimum concentration of Zn should be between 15 mg kg^−1^ and 20 mg kg^−1^ DW (Marschner, 1995). Mn is indispensable as it has an important role as a cofactor of various enzymes such as SOD and catalase and plays an important role in photosynthesis. For most plants, critical deficiency of Mn ranges between 10 and 20 mg Kg^−1^ DW (Marschner, 1995), and the threshold concentration for acute toxicity is 200 to 5,300 mg kg^−1^ DW (Edwards and Asher, 1982). In our investigation, it was observed that the maize treated with higher concentrations of EGC (1,000 mg L^−1^) contained high Cu concentrations (375.795 mg kg^−1^) and significantly lower concentrations of Zn and Mn (3.78 and 8.645 mg kg^−1^, respectively), lower than the optimum concentration required by the maize for normal growth. This explains the reduction in photosynthetic pigments and the overall reduction in biomass. Usually, it is seen that a high Cu level reduces the availability of Zn (Mousavi et al., 2012; Prasad et al., 2016). In concurrence with our study, Ivanova et al. (2010) reported that higher concentration of Cu negatively influenced the Zn uptake in rapeseed. A similar negative correlation between Zn and Cu in plants was also observed by Haldar and Mandal (1981) in rice. In agreement with our finding, antagonism between Mn and Cu was observed in *Brassica oleracea* L. var. *Botrytis* (Nautiyal and Chatterjee, 2007). Similarly, in a marine diatom (*Nitzschia closterium*), surface negative interaction of Cu and Mn was observed where Mn at higher concentration was found to alleviate the Cu toxicity (Stauber and Florence, 1985).

## 5. Conclusion

The present investigation demonstrated the EGC as a potential alternate fungicide to manage phytopathogens, particularly *B. maydis* at an optimum concentration (500 mg L^−1^). CuNPs and EO in combination showed synergistic effects against fungal pathogens of maize. The application of EGC led to increase in an enzymatic antioxidant activity and total phenol content in maize plants which strongly suggests that EGC could induce host defense mechanisms, alleviating the biotic stress. Our study also demonstrated the positive impact of EGC on different physiological parameters of maize crops at optimum concentrations of 250 and 500 mg L^−1^. Moreover, the phytotoxicity of EGC, CuNPs, and EO (Clove oil) at higher concentrations has also been established. Thus, the use of nanoformulations such as EGC at optimized concentration can be a boon in plant disease management with an eco-friendly and sustainable approach. As a future prospect, the transcriptomic analysis would give better insights into how the CuNPs and essential oil act on the pathogens. The study on nanoparticles' fate in the environment can be the next step to understanding their persistence with residual activity. More efforts need to be directed to develop safer nanoformulations and their cautious usage in sustainable agriculture.

## Data availability statement

The original contributions presented in the study are included in the article/Supplementary material, further inquiries can be directed to the corresponding authors.

## Author contributions

LD carried out the investigation, executed lab and net house experiments, generated data, and wrote the manuscript. RG was involved in the conceptualization, supervision, and writing and reviewing of the final draft. DK helped in data analysis and interpretation. RK supervised the synthesis part. TKM and SP contributed to the manuscript revision. BG helped in the statistical analysis of the data. All authors contributed to the article and approved the submitted version.
