# Supplementary material for: Essential oil-grafted copper nanoparticles as a potential next-generation fungicide for holistic disease management in maize
Source: Front Microbiol. 2023 Jul 6;14:1204512. doi: 10.3389/fmicb.2023.1204512 (PMC10361667; doi:10.3389/fmicb.2023.1204512)
Supplement: Supplementary file 1 [file Data_Sheet_1.docx]

**Materials and methods**

**Biochemical study of enzymatic antioxidant**

**Peroxidase (POX) assay**

A 100 mg of sample was grounded with 2 ml of 0.1 M Phosphate buffer (pH 7.5) in a pre-chilled mortar pestle. The homogenate obtained was subjected to centrifugation (13000 rpm for 35 min) and then the supernatant was collected in an Eppendorf tube (2 ml). A reaction mixture of 3 ml was prepared to contain 1, 0.5, and 0.5 ml of 100 mM phosphate buffer (pH 7.8), 96 mM Guaiacol, and 12 mM H_2_O_2,_ respectively, and the remaining volume was adjusted with H_2_O. A volume of 300 µl was dispensed in the well of the microplate reader and then 20 µl enzyme extract was added. Immediately, absorbance was recorded at 470 nm with 30 s intervals for 3 min using a spectrophotometer (EPOCH L2 microplate reader, Agilent Technologies, USA).

**Polyphenol oxidase (PPO) assay**

Homogenized 100 mg of leave sample with 2 ml of 0.1 M Phosphate buffer (pH 7.5) was centrifuged at 13000 rpm for 35 min and the supernatant was collected in 2 ml of Eppendorf tube. A 0.05 M Pyrogallol (300 μl) as a substrate prepared in 0.05 M Tris-HCl buffer (pH 7.0) solution, was first dispensed in the well and then 50 μl of the sample was added. Immediately, the absorbance was recorded at 420 nm with a 30 s interval for 3 min using a spectrophotometer (EPOCH L2 microplate reader, Agilent Technologies, USA).

***β-* 1, 3-glucanase assay**

Accurately weighed 0.5 g of sample, crushed, and ground into powder using liquid nitrogen. A 1.5 ml of 0.05 M potassium acetate buffer (pH 5.0) was added and homogenized the sample. The homogenate was then filtered using a sterilized muslin cloth followed by centrifugation at 14000 rpm for 10 min and the supernatant was collected which was further used as an enzyme extract. A 100 µl of enzyme extract was added immediately to 50 µl of 2 % laminarin and then incubated in a water bath at 50 °C for 2 h. The reaction was stopped by adding 300 µl of dinitrocyclic reagent. Then the reaction mixture was diluted 10 times with distilled H_2_O. Finally, using the spectrophotometer (Epoch L2 microplate reader, Agilent Technologies, USA), the absorbance was measured at 500 nm.

**Phenylalanine ammonia lyase (PAL) assay**

In a pre-chilled mortar and pestle, 0.5 g of leaf sample was ground using liquid nitrogen. One ml borate buffer (0.05 M) with pH 8.5 containing 20 % polyvinyl pyrrolidine (PVP) was added, homogenized, and centrifuged at 14000 rpm for 20 min at 4 °C. Supernatant was collected and used for further assay. For the assay, a 1.5 ml reaction mixture containing 0.5 ml of L-phenylalanine (4 mM), 0.75 ml of borate buffer (0.05 M, pH 8.5), 50 µl of enzyme extract, and 200 µl of H_2_O was incubated at 38 °C in a hot water bath for 1 h. On completion of the reaction, it was stopped with 0.1 ml of 5 M HCl. The absorbance of the sample was recorded at 290 nm using a spectrophotometer (EPOCH L2 microplate reader, Agilent Technologies, USA) to determine the amount of trans-cinnamic acid formed by the deamination of phenylalanine. A reaction mixture without phenylalanine was used as a control.

**Determination of total phenolic content (non-enzymatic antioxidant)**

A 100 g of sample was accurately weighed, crushed, and homogenized with pre-chilled 90% methanol in pre-chilled mortar and pestle followed by incubation at room temperature (RT) for 48 h. The homogenized tissue sample was then centrifuged at 13000 rpm for 5 min at RT, supernatant was collected for further assay. In a tube, 500 μl of diluted FC reagent in the ratio 1:4 (FC: distilled H_2_0 and 50 μl of the leaf extract were added, mixed properly, and left undisturbed at RT for 5 min. Next, 500 μl of 10% Na_2_CO_3_ was added, mixed thoroughly, and kept in the dark condition for 1 h. The microplate reader (EPOCH L2 microplate reader, Agilent Technologies, USA) was used to record the absorbance of the sample at 760 nm.

**Supplementary Figure 1** A Standard curve of D- glucose

**Supplementary Figure 2** A Standard curve of trans-cinnamic acid (CA)

**Supplementary Figure 3** A Standard curve of gallic acid

**Supplementary Table 1** Disease rating scale (1-9) of maydis leaf blight of maize (AICMIP, 2016)

| **Score** | **Degree of infection** | **PDI** | **Degree of reaction** |
| --- | --- | --- | --- |
| 1 | Nil to very slight infection (≤10%). | ≤11.11 | Resistant (R)  (Score: ≤ 3.0)  (PDI: ≤33.33) |
| 2 | Slight infection, a few lesions scattered on two lower leaves (10.1-20%). | 22.22 |  |
| 3 | Light infection, a moderate number of lesions scattered on four lower leaves (20.1-30%). | 33.33 |  |
| 4 | Light infection, a moderate number of lesions scattered on lower leaves, a few lesions scattered on middle leaves below the cob (30.1-40%). | 44.44 | Moderately resistant  (MR)  (Score: 3.1–5.0)  (PDI: 33,34-55.55) |
| 5 | Moderate infection, an abundant number of lesions scattered on lower leaves, and a moderate number of lesions scattered on middle leaves below the cob (40.1-50%). | 55.55 |  |
| 6 | Heavy infection, an abundant number of lesions scattered on lower leaves, moderate infection on middle leaves, and a few lesions on two leaves above the cob (50.1- 60%). | 66.66 | Mod. susceptible  (MS)  (Score: 5.1-7.0)  (PDI: 55.56-77.77) |
| 7 | Heavy infection, an abundant number of lesions scattered on lower and middle leaves, and a moderate number of lesions on two to four leaves above the cob (60.1-70%). | 77.77 |  |
| 8 | Very heavy infection, lesions abundant scattered on lower and middle leaves and Spreading up to the flag leaf (70.1-80%). | 88.88 | Susceptible (S)  (Score: >7.0)  (PDI: >77.77) |
| 9 | Very heavy infection, lesions abundant, scattered on almost all the leaves, plants prematurely dried and dead (>80%). | 99.99 |  |

**Supplementary Table 2** Peak intensity, d-spacing value, and miller indices for Essential oil grafted CuNPs

| **S.No.** | **2*θ* value** | **Standard 2*θ* (JCPDS 04-0836)** | **d-spacing value (Å)** | **Miller indices (*hkl*)** | **FWHM (Radians)** | **Crystallite Size (nm)** |
| --- | --- | --- | --- | --- | --- | --- |
| 1 | 43.299° | 43.297 | 2.039 | 111 | 0.2373 | 36.01868 |
| 2 | 50.437° | 50.433 | 1.750 | 200 | 0.31551 | 27.83184 |
| 3 | 74.139° | 74.130 | 1.191 | 220 | 0.41883 | 23.77216 |

**Supplementary Table 3** Efficacy of EO-grafted CuNPs against important maize fungal pathogens

| **S. No.** | **Treatments** | ***Bipolaris maydis*** | | ***Rhizoctonia solani* f.sp*. sasakii*** | | ***Fusarium verticillioides*** | |
| --- | --- | --- | --- | --- | --- | --- | --- |
|  |  | **Radial Growth (cm)*** | **Percent Inhibition (%)*** | **Radial Growth (cm)*** | **Percent Inhibition (%)*** | **Radial Growth (cm)** | **Percent Inhibition (%)** |
| 1 | Control | 5.000 | 0.000(0.00±0.000^)^#**^i^** | 7.000 | 0.000(0.000±0.000)**^i^** | 7.000 | 0.000(0.000±0.000)**^i^** |
| 2 | EGC @20 µg ml^-1^ | 4.067 | 20.000(25.559±0.995)**^g^** | 6.033 | 13.809(21.735±1.405)**^h^** | 5.566 | 20.476(26.891±0.337)**^g^** |
| 3 | EGC @40 µg ml^-1^ | 3.600 | 30.000(31.906±1.292)**^f^** | 4.5333 | 35.238(36.393±0.757)**^g^** | 4.533 | 35.238(36.399±0.286)**^f^** |
| 4 | EGC @60 µg ml^-1^ | 2.530 | 54.000(44.600±1.378)**^d^** | 2.033 | 70.952(57.366±0.300)**^d^** | 3.733 | 46.667(43.069±0.985)**^e^** |
| 5 | EGC @ 80 µg ml^-1^ | 1.467 | 62.000(57.366±3.330)**^b^** | 1.500 | 78.571(62.408±0.576)**^c^** | 3.100 | 55.714(48.265±0.825)**^d^** |
| 6 | EGC @100 µg ml^-1^ | 0.000 | 100.000(90.000±0.000)**^a^** | 0.000 | 100.000(90.000±0.000**^a^** | 1.734 | 75.238(60.172±1.374)**^c^** |
| 7 | EGC @120 µg ml^-1^ | 0.000 | 100.000(90.000±0.000)**^a^** | 0.000 | 100.000(90.000±0.000**^a^** | 0.000 | 100.000(0.000±0.000)^a^ |
| 8 | EGC @140 µg ml^-1^ | 0.000 | 100.000(90.000±0.000)**^a^** | 0.000 | 100.000(90.000±0.000**^a^** | 0.000 | 100.000(0.000±0.000)^a^ |
| 9 | CuNPs @100 µg ml^-1^ | 4.066 | 22.000(25.237±0.000)**^g^** | 4.200 | 40.000(39.209±0.965)**^f^** | 5.467 | 21.904(27.892±0.328)**^g^** |
| 10 | CuNPs @200 µg ml^-1^ | 2.267 | 52.000(47.662±1.016)**^d^** | 2.833 | 59.524(50.480±1.210)**^e^** | 3.267 | 53.333(46.894±0.985)**^d^** |
| 11 | EO @100 µg ml^-1^ | 3.867 | 24.000(28.400±0.923)**^fg^** | 4.633 | 33.809(35.152±0.765)**^g^** | 5.667 | 19.047(25.863±0.346)**^g^** |
| 12 | EO @200 µg ml^-1^ | 1.867 | 64.000(52.322±0.787)**^c^** | 2.633 | 62.381(52.151±0.744)**^e^** | 3.433 | 50.952(45.528±0.984)**^de^** |
| 13 | Fungicides @100 µg ml^-1^ | 4.633 | 4.000(15.461±2.048)**^h^** | 2.900 | 58.571(49.917±0.480)**^e^** | 2.033 | 70.952(57.407±1.605)**^c^** |
| 14 | Fungicides @200 µg ml^-1^ | 3.100 | 36.000(38.037±0.681)**^e^** | 1.2.00 | 82.857(65.563±1.258)**^b^** | 0.867 | 87.619(69.378±0.418)**^b^** |
| 15 | Tween-80 @1000 µg ml^-1^ | 5.000 | 0.000(0.000±0.000)**^i^** | 7.000 | 0.000(0.000±0.000)**^i^** | 6.933 | 0.952(3.243±3.243)**^h^** |
| 16 | DMSO @1000 µg ml^-1^ | 5.000 | 0.000(0.000±0.000)**^i^** | 7.000 | 0.000(0.000±0.000)**^i^** | 7.000 | 0.000(0.000±0.000)**^i^** |

*Data are the mean of three replications. ^#^Data within parentheses are Angular-transformed values. Value ± Standard errors followed by different letters in each column indicate a significant difference (Tukey HSD, *p*≤0.01). CuNPs: Copper nanoparticles, EGC= Essential oil-grafted CuNPs, EO: Essential oil (Clove oil), Fungicides: Mancozeb 75 % WP (for *Bipolaris maydis*), Carbendazim 50% WP (for *Rhizoctonia solani* f.sp. *sasakii* and *Fusarium verticillioides*)

**Supplementary Table 4** Efficacy of EO-grafted CuNPs against important maize fungal pathogens

| **S. No.** | **Treatments** | ***Macrophomina phaseolina*** | | ***Sclerotium rolfsii*** | |
| --- | --- | --- | --- | --- | --- |
|  |  | **Radial Growth (cm)*** | **Percent Inhibition (%)*** | **Radial Growth (cm)*** | **Percent Inhibition (%)*** |
| 1 | Control | 7 | 0.0000 (0.000±0.000)#**^h^** | 7 | 0.0000 (0.000±0.000)**^j^** |
| 2 | EGC @20 µg ml^-1^ | 6.133 | 12.381 (20.567±0.816)**^g^** | 4.966 | 29.048 (32.590±0.792) **^g^** |
| 3 | EGC @40 µg ml^-1^ | 5.333 | 23.809 (29.186±0.636)**^e^** | 4.367 | 37.619 (37.814±0.565)**^f^** |
| 4 | EGC @60 µg ml^-1^ | 4.766 | 31.904 (34.370±0.772)**^d^** | 4.000 | 42.857 (40.876±0.477) **^e^** |
| 5 | EGC @ 80 µg ml^-1^ | 3.333 | 52.381 (46.347±0.722)**^b^** | 3.266 | 53.333 (46.893±0.546)**^d^** |
| 6 | EGC @100 µg ml^-1^ | 0.000 | 100.000 (90.000±0.000)**^a^** | 2.833 | 59.523 (50.470±0.278)**^c^** |
| 7 | EGC @120 µg ml^-1^ | 0.000 | 100.000 (90.000±0.000)**^a^** | 2.000 | 71.429 (57.683±1.047)**^b^** |
| 8 | EGC @140 µg ml^-1^ | 0.000 | 100.000 (90.000±0.000)**^a^** | 0.000 | 100.000 (90.000±0.000)**^a^** |
| 9 | CuNPs @100 µg ml^-1^ | 0.000 | 18.095 (25.139±0.946)**^f^** | 5.300 | 24.286 (29.507±0.551)**^h^** |
| 10 | CuNPs @200 µg ml^-1^ | 5.733 | 46.667 (43.069±0.985)**^c^** | 2.966 | 57.619 (49.366±0.731)**^cd^** |
| 11 | EO @100 µg ml^-1^ | 3.733 | 16.190 (23.713±0.368)**^f^** | 5.133 | 26.666 (31.066±0.820)**^gh^** |
| 12 | EO @200 µg ml^-1^ | 5.867 | 47.619 (43.615±1.521)**^c^** | 2.266 | 67.619 (55.308±10.46)**^b^** |
| 13 | Fungicides @100 µg ml^-1^ | 3.666 | 53.333 (46.893±0.273)**^b^** | 3.000 | 57.143 (49.091±0.955)**^cd^** |
| 14 | Fungicides @200 µg ml^-1^ | 3.267 | 100.000 (90.000±0.000)**^a^** | 0.000 | 100.000 (90.000±0.000)**^a^** |
| 15 | Tween-80 @1000 µg ml^-1^ | 0.000 | 0.000 (0.000±0.000)**^h^** | 6.933 | 0.9524 (3.243±3.243)**^i^** |
| 16 | DMSO @1000 µg ml^-1^ | 7.000 | 0.000 (0.000±0.000)**^h^** | 7 | 0.000 (0.000±0.000)**^j^** |

*Data are the mean of three replications. ^#^Data within parentheses are Angular-transformed values. Value ± Standard errors followed by different letters in each column indicate a significant difference (Tukey HSD, *p*≤0.01). CuNPs: Copper nanoparticles, EGC= Essential oil-grafted CuNPs, EO: Essential oil (Clove oil), Fungicides: Carbendazim 50% WP (for *Macrophomina phaseolina*), Hexaconazole 5% SC (for *Sclerotium rolfsii*)

**Supplementary Table 5** *In-vivo* efficacy (Net house) of EO-grafted CuNPs and other treatments against maydis leaf blight of maize from May to Sep of 2021 (1^st^ Season)

| **S. No.** | | **Treatments** | **1^st^ scoring** | | **2^nd^ scoring** | |
| --- | --- | --- | --- | --- | --- | --- |
|  |  |  | ***Score**** | ***PDI (%)**** | ***Score**** | ***PDI (%)**** |
| 1 | ST (EGC) @250 mg L^-1^ | | 2.810±0.095**^c^** | 31.227(33.948±0.651)#**^cd^** | 2.952±0.048**^c^** | 32.804(34.927±0.323)**^cde^** |
| 2 | ST (EGC) @500 mg L^-1^ | | 2.667±0.095**^c^** | 29.639(32.959±0.660)**^cdef^** | 2.762±0.095**^c^** | 30.688(33.619±0.660)**^cdefg^** |
| 3 | ST (EGC) @1000 mg L^-1^ | | 2.286±0.165**^c^** | 25.397(30.221±1.208)**^efg^** | 2.381±0.126**^c^** | 26.455(30.925±0.914)**^fgh^** |
| 4 | ST (CuNPs) @1000 mg L^-1^ | | 2.571±0.082^c^ | 28.571(32.293±0.581)**^cdef^** | 2.714±0.165**^c^** | 30.158(33.277±1.145)**^defg^** |
| 5 | ST (EO) @100  mg L^-1^ | | 4.524±0.126^b^ | 50.265(45.133±0.802)**^b^** | 4.762±0.172**^b^** | 52.910(46.652±1.094)**^b^** |
| 6 | FS (EGC) @250 mg L^-1^ | | 3.095±0.095**^c^** | 34.392(35.886±0.636)**^c^** | 3.190±0.048^c^ | 35.450(36.525±0.316)**^c^** |
| 7 | FS (EGC) @500 mg L^-1^ | | 3.048±0.126**^c^** | 33.862(35.563±0.845)**^c^** | 3.143±0.082**^c^** | 34.921(36.206±0.551)**^cd^** |
| 8 | FS (EGC) @1000 mg L^-1^ | | 2.714±0.247**^c^** | 30.158(33.252±1.720)**^cde^** | 2.810±0.172**^c^** | 31.217(33.935±1.173)**^cdef^** |
| 9 | FS (CuNPs) @1000 mg L^-1^ | | 2.952±0.126**^c^** | 32.804(34.919±0.857)**^c^** | 3.048±0.095**^c^** | 33.862(35.566±0.643)**^cd^** |
| 10 | FS (EO) @1000 mg L^-1^ | | 4.952±0.126**^ab^** | 55.026(47.871±1.328)**^b^** | 5.058±0.126**^b^** | 56.085(48.478±0.809)**^b^** |
| 11 | ST+FS (EGC) @250 mg L^-1^ | | 2.429±0.082**^c^** | 26.984(31.277±0.591)**^def^** | 2.571±0.165**^c^** | 28.571(32.276±1.163)**^efg^** |
| 12 | ST+FS (EGC) @500 mg L^-1^ | | 2.333±0.095**^c^** | 25.925(30.587±0.696)**^defg^** | 2.429±0.143**^c^** | 26.984(31.264±1.035)**^efgh^** |
| 13 | ST+FS (EGC) @ mg L^-1^ | | 1.952±0.333**^c^** | 21.693(27.591±2.566)**^g^** | 2.143±0.247**^c^** | 23.809(29.120±1.857)**^h^** |
| 14 | ST+FS (CuNPs) @1000 mg L^-1^ | | 2.190±0.208**^c^** | 24.338(29.500±1.535)**^fg^** | 2.333±0.265**^c^** | 25.926(30.529±1.901)**^gh^** |
| 15 | ST+FS (EO) @1000 mg L^-1^ | | 4.476±0.048**^b^** | 49.735(44.830±0.303)**^b^** | 4.619±0.048**^b^** | 51.322(45.740±0.303)**^b^** |
| 16 | Absolute control (Water Spray) | | 0.095±0.095**^d^** | 1.058(3.420±3.420)**^h^** | 0.190±0.095**^d^** | 2.116(6.840±3.420)**^i^** |
| 17 | Manc @2000  mg L^-1^ | | 3.048±0.126**^c^** | 33.862(35.563±0.845)**^c^** | 3.238±0.126**^c^** | 35.978(36.835±0.838)**^c^** |
| 18 | NC (Pathogen only) | | 5.714±0.143**^a^** | 63.492(52.815±0.949)**^a^** | 6.048±0.172**^a^** | 67.196(55.052±1.158)**^a^** |

*Data are the mean of three replications. Data (Mean ± Standard errors) followed by different letters in each column indicate a significant difference (Tukey HSD, *p*≤0.05). #Data within the parentheses are Angular transformed values. Disease data scored twice on 20 and 30 DAI. (ST: Seed Treatment, FS: Foliar Spray, PDI: Percentage Disease index, EGC= EO grafted CuNPs, EO: Essential oil (Clove oil), Manc: Mancozeb 75 % WP, NC: Negative Control).

**Supplementary Table 6** *In-vivo* efficacy (Net house) of EO-grafted CuNPs and other treatments against maydis leaf blight of maize from May to Sep of 2022 (2^nd^ Season)

| **S. No.** | **Treatments** | **1^st^ scoring** | | **2^nd^ scoring** | |
| --- | --- | --- | --- | --- | --- |
|  |  | ***Score**** | ***PDI (%)**** | ***Score**** | ***PDI (%)**** |
| 1 | ST (EGC) @250 mg L^-1^ | 2.714±0.082**^cd^** | 30.159(33.292±0.572)**^ef^** | 3.000±0.082**^cd^** | 33.333(35.246±0.557)^e^ |
| 2 | ST (EGC) @500 mg L^-1^ | 2.571±0.082**^cde^** | 28.571 (32.293±0.581)**^fg^** | 2.810±0.126**^cde^** | 31.217(33.943±0.869)**^ef^** |
| 3 | ST (EGC) @1000 mg L^-1^ | 2.190±0.048**^de^** | 24.339 (29.546±0.352)**^h^** | 2.571±0.000**^cde^** | 28.571(32.299±0.000)**^fg^** |
| 4 | ST (CuNPs) @1000 mg L^-1^ | 2.667±0.126**^cde^** | 29.629 (32.953±0.882)**^ef^** | 2.905±0.048**^cde^** | 32.275(34.603±0.323)**^e^** |
| 5 | ST (EO) @100  mg L^-1^ | 4.476±0.126**^b^** | 49.735(44.830±0.802)**^c^** | 4.810±0.095**^b^** | 53.439(46.954±0.608)**^c^** |
| 6 | FS (EGC) @250 mg L^-1^ | 3.000±0.082**^cd^** | 33.333(35.246±0.557)**^de^** | 3.333±0.048**^c^** | 37.037(37.471±0.313)**^d^** |
| 7 | FS (EGC) @500 mg L^-1^ | 2.952±0.126**^cd^** | 32.804(34.919±0.857)**^def^** | 3.095±0.048**^cd^** | 34.39(35.889±0.320)**^de^** |
| 8 | FS (EGC) @1000 mg L^-1^ | 2.571±0.000**^cde^** | 28.571(32.299±0.000)**^fg^** | 2.905±0.048**^cde^** | 32.275(34.603±0.323)**^e^** |
| 9 | FS (CuNPs) @1000 mg L^-1^ | 2.762±0.048**^cd^** | 30.688(33.624±0.328)**^ef^** | 3.095±0.048**^cd^** | 34.391(35.889±0.320)**^de^** |
| 10 | FS (EO) @1000 mg L^-1^ | 5.190±0.208**^ab^** | 57.671(49.403±1.340)**^b^** | 5.286±0.218**^b^** | 58.730(50.019±1.407)**^de^** |
| 11 | ST+FS (EGC) @250 mg L^-1^ | 2.286±0.082**^cde^** | 25.397(30.242±0.603)**^gh^** | 2.476±0.048**^de^** | 27.513(31.622+0.338)**^g^** |
| 12 | ST+FS (EGC) @500 mg L^-1^ | 2.143±0.282**^de^** | 23.809(29.186±0.616)**^h^** | 2.429±0.082**^de^** | 26.280(31.277±0.591)**^g^** |
| 13 | ST+FS (EGC) @ mg L^-1^ | 1.762±0.048**^e^** | 19.577(26.246±0.380)**^i^** | 2.095±0.048**^e^** | 23.280(28.834±0.360)**^h^** |
| 14 | ST+FS (CuNPs) @1000 mg L^-1^ | 2.286±0.082**^cde^** | 25.396(30.242±0.603)**^gh^** | 2.381±0.048**^de^** | 26.455(30.939±0.345)**^g^** |
| 15 | ST+FS (EO) @1000 mg L^-1^ | 4.667±0.095**^b^** | 51.851(46.043±0.607)**^c^** | 4.810±0.126**^b^** | 53.439(46.955±0.804)**^f^** |
| 16 | Absolute control (Water Spray) | 0.048±0.048**^f^** | 0.529 (2.412±+2.211)^j^ | 0.143±0.000**^f^** | 1.587(7.235±0.000)**^i^** |
| 17 | Manc @2000  mg L^-1^ | 3.190±0.172**^c^** | 35.450 (36.512±1.148)**^d^** | 3.381±0.048**^c^** | 37.566(37.784±0.313)**^d^** |
| 18 | NC (Pathogen only) | 5.905±0.048**^a^** | 65.608 (54.074±0.320)**^a^** | 6.286±0.082**^a^** | 69.841(56.672±0.572)**^a^** |

*Data are the mean of three replications. Data (Mean ± Standard errors) followed by different letters in each column indicate a significant difference (Tukey HSD, *p*≤0.05). #Data within the parentheses are Angular transformed values. Disease data scored twice on 20 and 30 DAI. (ST: Seed Treatment, FS: Foliar Spray, PDI: Percentage Disease index, EGC= EO grafted CuNPs, EO: Essential oil (Clove oil), Manc: Mancozeb 75 % WP, NC: Negative Control).

**Supplementary Table 7** Effect of EO-grafted CuNPs and other treatments on *β*-1, 3-glucanase

| ***β*-1,3, Glucanse enzyme Activity (****µ mol g^-1^ FW)** | | | | | | | |
| --- | --- | --- | --- | --- | --- | --- | --- |
| S. N. | Treatments | Time Intervals | | | | | **Mean Treatments** |
|  |  | **0 h** | **24 h** | **48 h** | **72 h** | **96 h** |  |
| 1 | ST (EGC) @250 mg L^-1^ | 560.833 | 552.500 | 410.833 | 432.500 | 420.000 | 475.333 **^e^** |
| 2 | ST (EGC) @500 mg L^-1^ | 534.167 | 554.167 | 538.333 | 455.000 | 486.667 | 513.667 **^cde^** |
| 3 | FS (EGC) @250 mg L^-1^ | 487.500 | 549.167 | 550.833 | 470.000 | 468.333 | 505.167 **^de^** |
| 4 | FS (EGC) @500 mg L^-1^ | 484.167 | 560.000 | 595.833 | 599.167 | 537.500 | 555.333 **^bc^** |
| 5 | ST+FS (EGC) @250 mg L^-1^ | 528.333 | 620.000 | 600.833 | 570.833 | 532.500 | 570.500 **^b^** |
| 6 | ST+FS (EGC) @500 mg L^-1^ | 565.833 | 625.000 | 623.333 | 754.167 | 809.167 | 675.500 **^a^** |
| 7 | Absolute control (Water Spray) | 392.500 | 386.667 | 370.833 | 334.167 | 365.000 | 369.833 **^g^** |
| 8 | Manc @2000 mg L^-1^ | 485.000 | 340.833 | 473.333 | 395.833 | 406.667 | 420.333 **^f^** |
| 9 | NC (Pathogen only) | 713.333 | 578.333 | 609.167 | 397.500 | 455.000 | 550.667 **^bcd^** |
|  | **Mean (Time intervals)** | 527.963 **^ab^** | 529.630 **^a^** | 530.370 **^a^** | 489.907 **^c^** | 497.870 **^bc^** |  |

Data are the mean of three replications. Data (Mean ± Standard errors) followed by different letters in each column indicate a significant difference (Tukey HSD, *p*≤0.05). Maize leaves were collected five days after spraying. ST: Seed Treatment, FS: Foliar Spray, EGC: EO-grafted CuNPs, EO: Essential oil (Clove oil), Manc: Mancozeb 75 % WP, NC: Negative Control

**Supplementary Table 8** Effect of EGC and other treatments on Phenylalanine ammonia Lyase (PAL)

| **PAL enzyme Activity (µg g^-1^)** | | | | | | | |
| --- | --- | --- | --- | --- | --- | --- | --- |
| S. N. | Treatments | Time Intervals | | | | | **Mean Treatments** |
|  |  | **0 h** | **24 h** | **48 h** | **72 h** | **96 h** |  |
| 1 | ST (EGC) @250 mg L^-1^ | 755.125 | 699.708 | 725.125 | 889.708 | 530.958 | 720.125**^ab^** |
| 2 | ST (EGC) @500 mg L^-1^ | 734.708 | 690.958 | 591.375 | 677.208 | 482.208 | 635.292**^bc^** |
| 3 | FS (EGC) @250 mg L^-1^ | 344.125 | 677.208 | 583.042 | 758.875 | 646.792 | 602.008**^c^** |
| 4 | FS (EGC) @500 mg L^-1^ | 328.875 | 343.458 | 740.958 | 592.208 | 335.958 | 468.292**^d^** |
| 5 | ST+FS (EGC) @250 mg L^-1^ | 760.125 | 799.708 | 608.875 | 995.542 | 798.458 | 792.542**^a^** |
| 6 | ST+FS (EGC) @500 mg L^-1^ | 668.042 | 667.625 | 730.542 | 555.125 | 428.458 | 609.958**^bc^** |
| 7 | Absolute control (Water Spray) | 258.042 | 244.708 | 246.375 | 231.792 | 292.625 | 254.708**^f^** |
| 8 | Manc @2000 mg L^-1^ | 350.542 | 332.208 | 312.625 | 343.042 | 334.708 | 334.625**^ef^** |
| 9 | NC (Pathogen only) | 345.958 | 370.125 | 405.792 | 473.458 | 468.875 | 412.842**^de^** |
|  | **Mean (Time intervals)** | 505.060**^b^** | 536.190**^b^** | 549.412^ab^ | 612.995**^a^** | 479.894**^b^** |  |

Data are the mean of three replications. Data (Mean ± Standard errors) followed by different letters in each column indicate a significant difference (Tukey HSD, *p*≤0.05). Maize leaves were collected five days after spraying. ST: Seed Treatment, FS: Foliar Spray, EGC: Essential oil-grafted CuNPs, EO: Essential oil (Clove oil), Manc: Mancozeb 75 % WP, NC: Negative Control

**Supplementary Table 9** Effect of EO-grafted CuNPs and other treatments on Peroxidase (POX)

| **POX enzyme Activity (µM min^-1^ g^-1^ FW)** | | | | | | | |
| --- | --- | --- | --- | --- | --- | --- | --- |
| S. N. | Treatments | Time Intervals | | | | | **Mean Treatments** |
|  |  | **0 h** | **24 h** | **48 h** | **72 h** | **96 h** |  |
| 1 | ST (EGC) @250 mg L^-1^ | 0.011 | 0.018 | 0.020 | 0.020 | 0.019 | 0.018**^b^** |
| 2 | ST (EGC) @500 mg L^-1^ | 0.013 | 0.016 | 0.011 | 0.012 | 0.020 | 0.014**^cd^** |
| 3 | FS (EGC) @250 mg L^-1^ | 0.013 | 0.014 | 0.021 | 0.013 | 0.019 | 0.016**^bc^** |
| 4 | FS (EGC) @500 mg L^-1^ | 0.014 | 0.020 | 0.009 | 0.010 | 0.005 | 0.012**^ef^** |
| 5 | ST+FS (EGC) @250 mg L^-1^ | 0.013 | 0.022 | 0.032 | 0.025 | 0.027 | 0.024**^a^** |
| 6 | ST+FS (EGC) @500 mg L^-1^ | 0.012 | 0.006 | 0.008 | 0.007 | 0.006 | 0.008**^g^** |
| 7 | Absolute control (Water Spray) | 0.009 | 0.010 | 0.010 | 0.011 | 0.008 | 0.010**^fg^** |
| 8 | Manc @2000 mg L^-1^ | 0.008 | 0.008 | 0.011 | 0.009 | 0.007 | 0.008**^g^** |
| 9 | NC (Pathogen only) | 0.009 | 0.015 | 0.013 | 0.012 | 0.017 | 0.013**^de^** |
|  | **Mean (Time intervals)** | 0.011**^c^** | 0.014**^ab^** | 0.015^a^ | 0.013**^b^** | 0.014**^ab^** |  |

Data are the mean of three replications. Data (Mean ± Standard errors) followed by different letters in each column indicate a significant difference (Tukey HSD, *p*≤0.05). Maize leaves were collected five days after spraying. ST: Seed Treatment, FS: Foliar Spray, EGC: Essential oil-grafted CuNPs, EO: Essential oil (Clove oil), Manc: Mancozeb 75 % WP, NC: Negative Control

**Supplementary Table 10** Effect of EO-grafted CuNPs and other treatments on Polyphenol oxidase (PPO)

| **PPO enzyme Activity (µM min^-1^ g^-1^ FW)** | | | | | | | |
| --- | --- | --- | --- | --- | --- | --- | --- |
| S. N. | Treatments | Time Intervals | | | | | **Mean Treatments** |
|  |  | **0 h** | **24 h** | **48 h** | **72 h** | **96 h** |  |
| 1 | ST (EGC) @250 mg L^-1^ | 0.238 | 0.230 | 0.233 | 0.205 | 0.214 | 0.224**^a^** |
| 2 | ST (EGC) @500 mg L^-1^ | 0.213 | 0.203 | 0.218 | 0.220 | 0.209 | 0.213**^a^** |
| 3 | FS (EGC) @250 mg L^-1^ | 0.188 | 0.233 | 0.233 | 0.196 | 0.192 | 0.208**^ab^** |
| 4 | FS (EGC) @500 mg L^-1^ | 0.183 | 0.221 | 0.191 | 0.195 | 0.188 | 0.196**^bc^** |
| 5 | ST+FS (EGC) @250 mg L^-1^ | 0.200 | 0.225 | 0.254 | 0.226 | 0.206 | 0.222**^a^** |
| 6 | ST+FS (EGC) @500 mg L^-1^ | 0.235 | 0.209 | 0.200 | 0.194 | 0.197 | 0.207**^ab^** |
| 7 | Absolute control (Water Spray) | 0.164 | 0.174 | 0.172 | 0.177 | 0.175 | 0.172**^c^** |
| 8 | Manc @2000 mg L^-1^ | 0.190 | 0.178 | 0.170 | 0.183 | 0.171 | 0.178**^c^** |
| 9 | NC (Pathogen only) | 0.207 | 0.195 | 0.221 | 0.205 | 0.187 | 0.203**^ab^** |
|  | **Mean (Time intervals)** | 0.202**^ab^** | 0.208**^ab^** | 0.210**^a^** | 0.200**^ab^** | 0.193**^b^** |  |

Data are the mean of three replications. Data (Mean ± Standard errors) followed by different letters in each column indicate a significant difference (Tukey HSD, *p*≤0.05). Maize leaves were collected five days after spraying. ST: Seed Treatment, FS: Foliar Spray, EGC: Essential oil-grafted CuNPs, EO: Essential oil (Clove oil), Manc: Mancozeb 75 % WP, NC: Negative Control

**Supplementary Table. 11** Effect of EGC and other treatments on Total Phenolics

| **Total Phenolics (µg GAE g^-1^ FW)** | | | | | | | |
| --- | --- | --- | --- | --- | --- | --- | --- |
| S. N. | Treatments | Time Intervals | | | | | **Mean Treatments** |
|  |  | **0 h** | **24 h** | **48 h** | **72 h** | **96 h** |  |
| 1 | ST (EGC) @250 mg L^-1^ | 25.035 | 24.273 | 29.289 | 31.469 | 30.961 | 28.205**^e^** |
| 2 | ST (EGC) @500 mg L^-1^ | 29.606 | 32.696 | 30.834 | 33.141 | 31.575 | 31.570**^c^** |
| 3 | FS (EGC) @250 mg L^-1^ | 20.548 | 26.834 | 39.003 | 35.680 | 31.024 | 30.618**^cd^** |
| 4 | FS (EGC) @500 mg L^-1^ | 20.442 | 34.284 | 35.130 | 29.437 | 28.294 | 29.517**^d^** |
| 5 | ST+FS (EGC) @250 mg L^-1^ | 21.331 | 38.262 | 48.273 | 48.781 | 37.670 | 38.863**^a^** |
| 6 | ST+FS (EGC) @500 mg L^-1^ | 22.268 | 48.146 | 48.130 | 32.871 | 26.712 | 35.625**^b^** |
| 7 | Absolute control (Water Spray) | 17.691 | 17.458 | 19.130 | 19.934 | 16.633 | 18.169**^h^** |
| 8 | Manc @2000 mg L^-1^ | 19.490 | 19.024 | 22.961 | 19.384 | 22.368 | 20.646**^g^** |
| 9 | NC (Pathogen only) | 21.945 | 26.432 | 25.183 | 26.199 | 24.337 | 24.819**^f^** |
|  | **Mean (Time intervals)** | 23.262**^d^** | 28.823**^c^** | 32.548**^a^** | 30.655**^b^** | 28.064**^c^** |  |

Data are the mean of three replications. Data (Mean ± Standard errors) followed by different letters in each column indicate a significant difference (Tukey HSD, *p*≤0.05). Maize leaves were collected five days after spraying. ST: Seed Treatment, FS: Foliar Spray, EGC: Essential oil-grafted CuNPs, EO: Essential oil (Clove oil), Manc: Mancozeb 75 % WP, NC: Negative Control

**Supplementary Table 12** Concentration-dependent effect of different treatments on maize plants

| **S.N** | **Treatment** | **Root length (m)** | **Root length Density (cm cm^-1^)** | **Root Volume (cm^3^)** |
| --- | --- | --- | --- | --- |
| 1 | Control | 35.598±1.696 b | 0.276±0.013 ab | 6.832±0.610 b |
| 2 | EGC @250 mg L^-1^ | 60.203±0.279 a | 0.467±0.002 a | 12.251±0.155 a |
| 3 | EGC @500 mg L^-1^ | 63.896±2.742 a | 0.495±0.021 a | 13.167±0.299 a |
| 4 | EGC @1000 mg L^-1^ | 30.173±0.050 b | 0.234±0.001 ab | 4.316±0.279 c |
| 5 | CuNPs @1000 mg L^-1^ | 33.158±0.098 b | 0.257±0.000 ab | 7.771±0.046 b |
| 6 | EO @1000 mg L^-1^ | 8.226±0.115 c | 0.064±0.001 b | 1.736±0.022 d |
| **S.N** | **Treatment** | **Average Diameter (mm)** | **Root Biomass**  **(g plant^-1^)** | **Shoot Biomass (g plant^-1^)** |
| 1 | Control | 0.497±0.015 a | 1.008±0.036 ab | 14.770±0.257 b |
| 2 | EGC @250 mg L^-1^ | 0.512±0.014 a | 1.534±0.130 a | 19.103±0.762 a |
| 3 | EGC @500 mg L^-1^ | 0.524±0.043 a | 1.611±0.063 a | 20.293±0.550 a |
| 4 | EGC @1000 mg L^-1^ | 0.489±0.046 a | 0.456±0.026 bc | 12.180±0.193 c |
| 5 | CuNPs @1000 mg L^-1^ | 0.496±0.039 a | 0.847±0.092 bc | 13.700±1.148 bc |
| 6 | EO @1000 mg L^-1^ | 0.391±0.015 a | 0.203±0.015 c | 7.110±0.263 d |
| **S.N** | **Treatment** | **Root Shoot Ratio** | **Germination Percentage** | **Vigor Index** |
| 1 | Control | 0.497±0.015 a | 95.238±4.762 b | 8799.206±25.392 c |
| 2 | EGC @250 mg L^-1^ | 0.512±0.014 a | 100.000±0.000 a | 9692.223±13.537 b |
| 3 | EGC @500 mg L^-1^ | 0.524±0.043 a | 90.476±4.762 c | 9817.618±46.068 a |
| 4 | EGC @1000 mg L^-1^ | 0.489±0.046 a | 66.667±4.762 e | 4787.143±32.700 e |
| 5 | CuNPs @1000 mg L^-1^ | 0.496±0.039 a | 80.952±4.762 d | 6623.809±34.818 d |
| 6 | EO @1000 mg L^-1^ | 0.391±0.015 a | 47.619±4.762 f | 2366.984±34.818 f |

Data are the mean of three replications. Data (Mean ± Standard errors) followed by different letters in each column indicate a significant difference (Tukey HSD, *p*≤0.05). ST: Seed Treatment, FS: Foliar Spray, EGC= EO-grafted CuNPs, CuNPs: Copper nanoparticles, EO: Essential oil (Clove oil**)**

**Supplementary Figure 4** Scanned roots of maize using WinRHIZO professional software (LA2400, Regent instrument, Quebec, Canada) showing the effect of different treatments


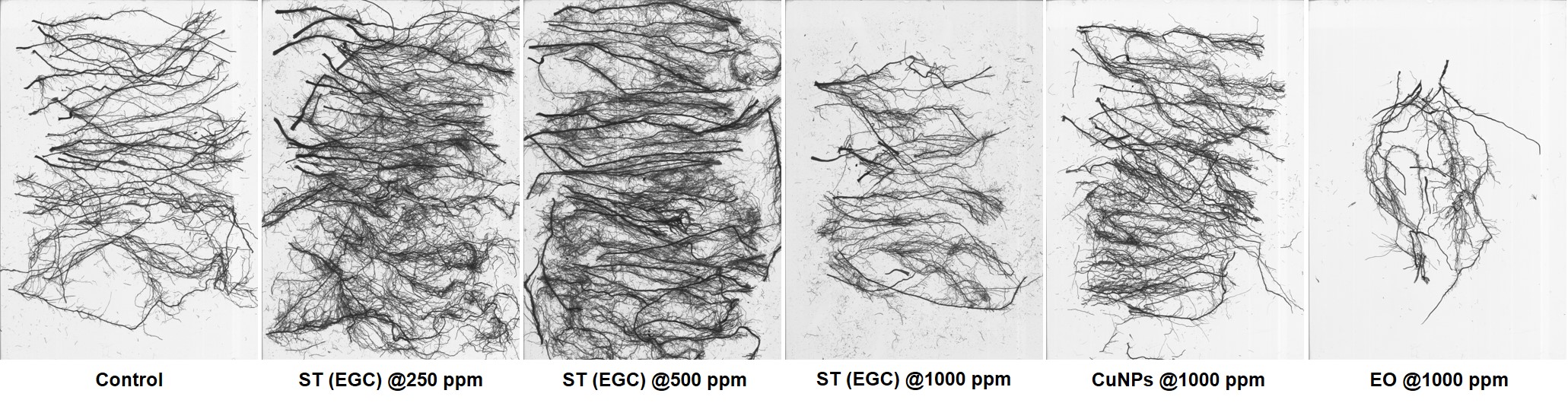


**Supplementary Table. 13** Effect of EO-grafted CuNPs and other treatments on photosynthetic pigments of maize leaves

| **S.No.** | **Treatments** | **Chlorophyll *a***  **(mg g^-1^ Fresh weight)** | **Chlorophyll *b***  **(mg g^-1^ Fresh weight)** | **Total Chlorophyll**  **(mg g^-1^ Fresh weight)** | **Total carotenoids (µg g^-1^ Fresh weight)** |
| --- | --- | --- | --- | --- | --- |
| 1 | ST (EGC) @250 mg L^-1^ | 2.264±0.007**^a^** | 0.515±0.034**^abc^** | 2.790±0.040**^a^** | 0.706**±**0.026**^a^** |
| 2 | ST (EGC) @500 mg L^-1^ | 2.056±0.019**^ab^** | 0.262±0.006**^cd^** | 2.326±0.017**^c^** | 0.611**±**0.022**^a^** |
| 3 | ST (EGC) @1000 mg L^-1^ | 1.047±0.005**^h^** | 0.258±0.005**^cd^** | 1.310±0.003**^g^** | 0.570**±**0.015**^a^** |
| 4 | ST (CuNPs) @1000 mg L^-1^ | 1.105±0.003**^gh^** | 0.498±0.007**^abc^** | 1.611±0.006**^ef^** | 0.482**±**0.065**^a^** |
| 5 | ST (EO) @100  mg L^-1^ | 1.070±0.012**^h^** | 0.593±0.005**^a^** | 1.671±0.015**^ef^** | 0.559**±**0.011**^a^** |
| 6 | FS (EGC) @250 mg L^-1^ | 1.475±0.003**^ef^** | 0.349±0.014**^abcd^** | 1.832±0.013**^de^** | 0.575**±**0.021**^a^** |
| 7 | FS (EGC) @500 mg L^-1^ | 1.311±0.002**^fg^** | 0.302±0.004**^bcd^** | 1.620±0.003**^ef^** | 0.517**±**0.008**^a^** |
| 8 | FS (EGC) @1000 mg L^-1^ | 1.107±0.008**^gh^** | 0.334±0.008**^abcd^** | 1.448±0.012**^fg^** | 0.489**±**0.002**^a^** |
| 9 | FS (CuNPs) @1000 mg L^-1^ | 0.968±0.004**^h^** | 0.369±0.005**^abcd^** | 1.344±0.008**^g^** | 0.473**±**0.004**^a^** |
| 10 | FS (EO) @1000 mg L^-1^ | 1.550±0.005**^de^** | 0.195±0.007**^d^** | 1.751±0.008**^e^** | 0.648**±**0.004**^a^** |
| 11 | ST+FS (EGC) @250 mg L^-1^ | 2.086±0.007**^ab^** | 0.539±0.011**^ab^** | 2.636±0.008**^ab^** | 0.681**±**0.003**^a^** |
| 12 | ST+FS (EGC) @500 mg L^-1^ | 2.232±0.011**^a^** | 0.090±0.015**^d^** | 2.330±0.008**^c^** | 0.593**±**0.012**^a^** |
| 13 | ST+FS (EGC) @ 1000 mg L^-1^ | 0.951±0.003**^h^** | 0.270±0.012**^bcd^** | 1.226 ±0.009**^g^** | 0.345**±**0.003**^a^** |
| 14 | ST+FS (CuNPs) @1000 mg L^-1^ | 1.049±0.011**^h^** | 0.245±0.015**^cd^** | 1.300±0.006**^g^** | 0.474**±**0.010**^a^** |
| 15 | ST+FS (EO) @1000 mg L^-1^ | 1.083±0.009**^gh^** | 0.523±0.006**^abc^** | 1.614±0.009**^ef^** | 0.533**±**0.002**^a^** |
| 16 | Absolute control (Water Spray) | 1.930±0.004**^bc^** | 0.485±0.014**^abc^** | 2.426±0.013**^bc^** | 0.705**±**0.004**^a^** |
| 17 | Manc @2000  mg L^-1^ | 1.923±0.014**^bc^** | 0.508±0.010**^abc^** | 2.441±0.010**^bc^** | 0.609**±**0.003**^a^** |
| 18 | NC (Pathogen only) | 1.780±0.003**^cd^** | 0.263±0.010**^cd^** | 2.051**±**0.013**^d^** | 0.636**±**0.003**^a^** |

*Data are the mean of three replications. Data (Mean ± Standard errors) followed by different letters in each column indicate a significant difference (Tukey HSD, *p*P≤0.05). ST: Seed Treatment, FS: Foliar Spray, EGC: EO-grafted CuNPs, CuNPs: Copper nanoparticles, EO: Essential oil (Clove oil)
